# Training Signaling Pathway Maps to Biochemical Data with Constrained Fuzzy Logic: Quantitative Analysis of Liver Cell Responses to Inflammatory Stimuli

**DOI:** 10.1371/journal.pcbi.1001099

**Published:** 2011-03-03

**Authors:** Melody K. Morris, Julio Saez-Rodriguez, David C. Clarke, Peter K. Sorger, Douglas A. Lauffenburger

**Affiliations:** 1 Center for Cell Decision Processes, Massachusetts Institute of Technology and Harvard Medical School, Boston, Massachusetts, United States of America; 2 Department of Biological Engineering, Massachusetts Institute of Technology, Cambridge, Massachusetts, United States of America; 3 Department of Systems Biology, Harvard Medical School, Boston, Massachusetts, United States of America; Medical College of Wisconsin, United States of America

## Abstract

Predictive understanding of cell signaling network operation based on general prior knowledge but consistent with empirical data in a specific environmental context is a current challenge in computational biology. Recent work has demonstrated that Boolean logic can be used to create context-specific network models by training proteomic pathway maps to dedicated biochemical data; however, the Boolean formalism is restricted to characterizing protein species as either fully active or inactive. To advance beyond this limitation, we propose a novel form of fuzzy logic sufficiently flexible to model quantitative data but also sufficiently simple to efficiently construct models by training pathway maps on dedicated experimental measurements. Our new approach, termed constrained fuzzy logic (cFL), converts a prior knowledge network (obtained from literature or interactome databases) into a computable model that describes graded values of protein activation across multiple pathways. We train a cFL-converted network to experimental data describing hepatocytic protein activation by inflammatory cytokines and demonstrate the application of the resultant trained models for three important purposes: (a) generating experimentally testable biological hypotheses concerning pathway crosstalk, (b) establishing capability for quantitative prediction of protein activity, and (c) prediction and understanding of the cytokine release phenotypic response. Our methodology systematically and quantitatively trains a protein pathway map summarizing curated literature to context-specific biochemical data. This process generates a computable model yielding successful prediction of new test data and offering biological insight into complex datasets that are difficult to fully analyze by intuition alone.

## Author Summary

Over the past few years, many methods have been developed to construct large-scale networks from the literature or databases of genetic and physical interactions. With the advent of high-throughput biochemical methods, it is also possible to measure the states and activities of many proteins in these biochemical networks under different conditions of cellular stimulation and perturbation. Here we use constrained fuzzy logic to systematically compare interaction networks to experimental data. This systematic comparison elucidates interactions that were theoretically possible but not actually operating in the biological system of interest, as well as data that was not described by interactions in the prior knowledge network, pointing to a need to increase our knowledge in specific parts of the network. Furthermore, the result of this comparison is a trained, quantitative model that can be used to make *a priori* quantitative predictions about how the cellular protein network will respond in conditions not initially tested.

## Introduction

Signaling networks regulate cell phenotypic responses to stimuli present in the extracellular environment [1]. High throughput “interactome” data provide critical information on the composition of these networks [2], [3], [4], but understanding their operation as signal processing systems is strongly advanced by direct interface with dedicated experimental data representing measured responses of biochemical species in the network (proteins, mRNA, miRNA, etc.) to stimulation by environmental cues in the presence or absence of perturbation [5], [6], [7], [8]. Immediate early responses are dominated by protein post-translational modifications (we focus here on phosphorylation), assembly of multi-protein complexes, and changes in protein stability and localization. Such responses are typically highly context dependent, varying with cell type and biological environment. A critical question for the field is how large scale measurements of these responses can be combined with a signed, directed protein signaling network (PSN) to better understand the operation of complex biochemical systems [9].

PSNs are typically deduced by manual or automated annotation of the literature (*e.g.* [10]) or directly from high-throughput experimental data (*e.g.* [11],[12],[13]) using a variety of computational techniques. PSNs are represented as node-edge graphs [14], and although they provide high-level insight into the composition and topology of regulatory networks [15], [16], [17], [18], [19], [20], as currently constituted PSNs are not readily ‘computable’ in that they cannot be used to calculate activation states of the key proteins in a pathway given a set of input cues, nor can quantitative relationships between pathways be determined. This restricts the utility of PSNs for explicit prediction of responses and makes it difficult to compare network representations to functional experimental data. A chief motivation of our current work is to determine how information encoded in a PSN can be made computable and compared to experimental data from a specific cell type, resulting in a context-specific network model.

Logic-based models (*e.g.* [21], [22], [23], [24], [25], [26]; reviewed in [27], [28]) offer one means for converting interaction maps into computable models. We have previously used Boolean logic (BL) to convert a literature-derived signed, directed PSN (comprising for this purpose a ‘prior knowledge network’ [PKN]) into a computable model that could be compared to experimental data consisting largely of the phospho-states of signal transduction proteins in the presence of different ligands and drugs [29]. This approach allowed us to determine which links in the PKN were supported by the data, and generated models that were useful in making predictions about network topology [29] and drug targets [30]. However, Boolean logic has a significant limitation, since real biochemical interactions rarely have simple on-off characteristics assumed by Boolean logic. Thus, we require a means to encode graded responses and typical sigmoidal biological relationships in a logic-based framework.

One way to accomplish this is to apply traditional fuzzy logic [FL], as demonstrated previously in modeling continuous input-output relationships to encode a complex signaling network [31], [32]. In the realm of control theory, FL modeling is an established technique for predicting the outputs of complex industrial processes when the influences of inputs cannot be characterized precisely [33], [34], [35]. A central feature of FL is that it accounts for graded values of process states using a virtually unlimited repertoire of relationships between model species or components. However, for past application to biochemical signaling networks, the flexibility of conventional FL modeling necessitated that the network topology be fixed prior to either manual [31] or computational [32] parameter fitting, rendering a formal training of network topology to experimental data infeasible.

In this paper we develop and employ a new approach to fuzzy logic modeling of biological networks that we term ‘constrained fuzzy logic’ [cFL] for descriptive purposes. A key feature of cFL modeling is that it limits the repertoire of relationships between model species, enabling the formal training of a PKN to experimental data and resulting in a quantitative network model. To maximize broad dissemination across the computational biology community, we implement cFL in an exisiting software tool CellNetOptimizer v2.0 (CellNOpt), significantly extended to accommodate the further requirements of cFL while maintaining the BL analytic approach (freely available at http://www.ebi.ac.uk/saezrodriguez/software.html). We demonstrate the value of the CellNOpt-cFL method by elucidating new information from a recently published experimental dataset describing phospho-protein signaling in HepG2 cells exposed to a set of inflammatory cytokines [36]. We show that a cFL model can be trained against a dataset and then validated by successful *a priori* prediction of test data absent from the training data. We also establish the benefits of cFL relative to BL in three key areas: (a) generation of new biological understanding; (b) quantitative prediction of signaling nodes; and (c) modeling quantitative relationships between signaling and cytokine release nodes. Particular examples of validated biological predictions include: (i) TGFα-induced partial activation of the JNK pathway and (ii) IL6-induced partial activation of multiple unexpected downstream species via the MEK pathway. Our work demonstrates the technical feasibility of cFL in modeling real biological data and generating new biological insights concerning the operation of canonical signaling networks in specific cellular contexts.

## Results

### Constraining fuzzy logic

Fuzzy logic is a highly flexible methodology to transform linguistic observations into quantitative specification of how the output of a gate depends on the values of the inputs [33], [37], [38], [39]. For example, in the simplest, ‘Sugeno’ form of fuzzy logic, one specifies the following: ‘membership functions’ designating a variable number of discrete categories (“low, medium, high', etc.) as well as what quantitative value of a particular input belongs either wholly or partially to these categories; ‘rules’ designating the logical relationships between the gate inputs and outputs; AND and OR ‘methods’ designating the mathematical execution of each logical relationship; ‘weights’ designating the credence given any rule; and ‘defuzzification’ designating a scheme for determining a final output value from the evaluation of multiple rules [40]. This flexibility is important in industrial process control [41], which aims to use uncertain and subjective linguistic terms to predict how a controller should modulate a process variable to achieve the desired output.

However, our goal is to train models on quantitative biological data that are inevitably incomplete in the sense that (i) measurements are not obtained under all possible conditions and (ii) available data are not sufficient to constrain both the topology and quantitative parameters of the underlying networks. Accordingly, we sought to develop a fuzzy logic system that minimizes the number of parameters to avoid over-fitting and simplifies the logic structure to facilitate model interpretability. Because we aim to represent relationships among proteins in enzymatic cascades, mathematical relationships should be biologically relevant. We therefore use a simple Sugeno fuzzy logic gate with a defined form (see Text S1) based on transfer functions (mathematical functions describing the relationship between input and output node values) that approximate the Hill functions of classical enzymology.

**Figure 1:**
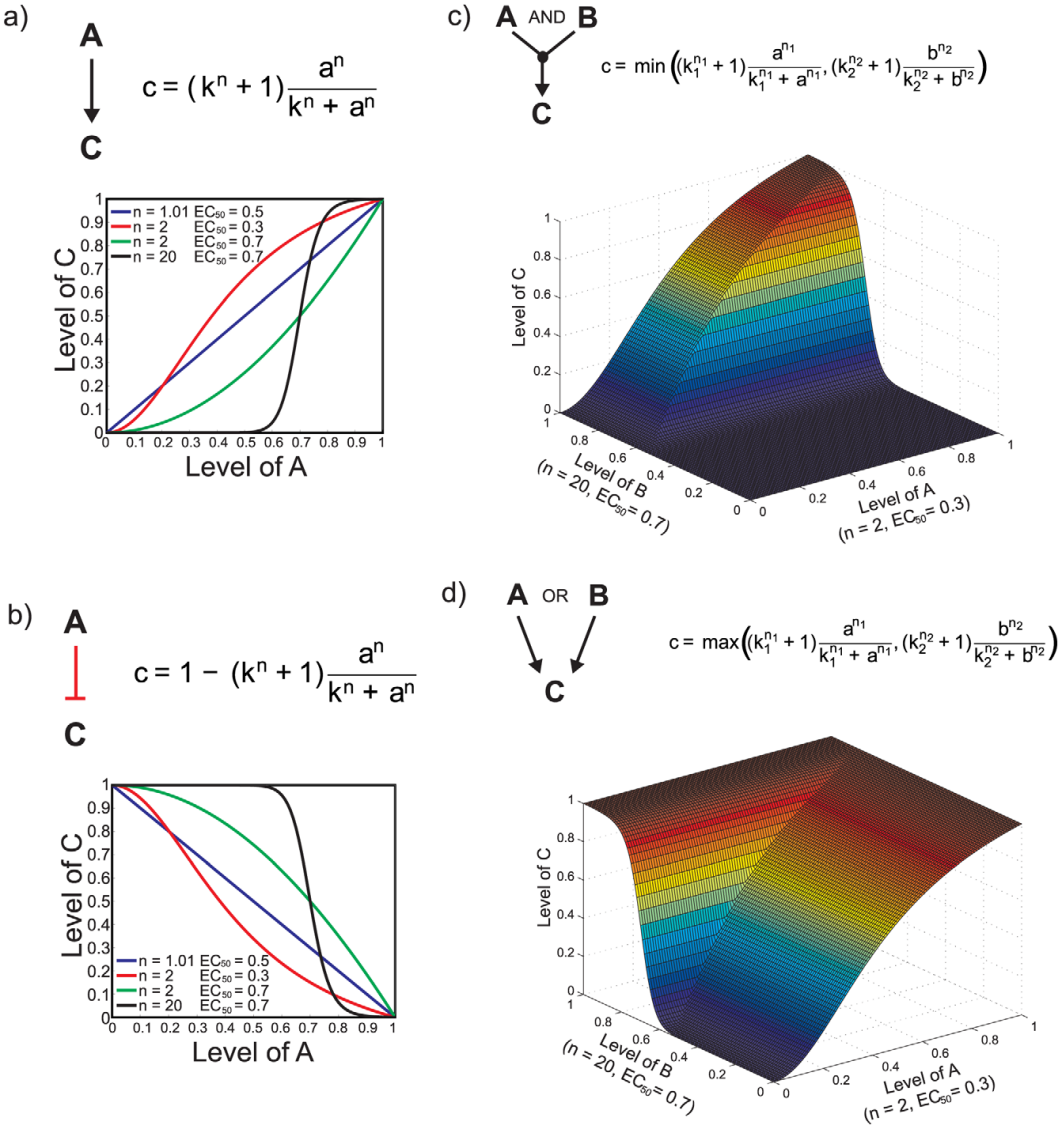
Construction of gates with constrained fuzzy logic (cFL). When node C depends only on node A, a normalized Hill function is used to calculate value of node C, ‘*c*’, given value of node A, ‘*a*’, where *n* is Hill coefficient and *k* is the sensitivity parameter specifying the EC_50_ for each gate. Several representative normalized Hill functions are shown for activating (a) and inhibiting (b) cFL gates. When C has more than one input (A and B, in this case), either an AND (c) or OR (d) gate must be used to model the interaction. In the case of the AND gate, the minimum possible value of *c* calculated from the transfer functions is used as the output node value. One possible response surface for levels of C given different levels of A and B with two transfer functions is demonstrated (c). For evaluation of an OR gate, the maximum value of *c* is used as the output node value, with the corresponding response surface (d).

Our ‘constrained’ fuzzy logic (cFL) framework uses a simplified fuzzy logic gate that is best described by the mathematical representation in Figure 1. The value of an output node of a one-input positive interaction is evaluated using a transfer function. In this paper ‘input-output’ refers to the nodes of a specific cFL logic gate, where ‘nodes’ are molecular species. We use the terms ‘model inputs’ and ‘model outputs’ to denote the overall relationship between model inputs such as ligand stimulation of cells and the collective output of the network (protein modifications or phenotypic states in our application). The transfer function underlying cFL gates is a normalized Hill function with two parameters: (1) the Hill coefficient, *n*, which determines the sharpness of the sigmoidal transition between high and low output node values and (2) the sensitivity parameter, *k*, which determines the midpoint of the function (corresponding to the EC_50_ value in a dose-response curve, Figure 1a). A negative interaction is represented similarly, except that the transfer function is subtracted from one, effectively inverting it (Figure 1b). Varying these parameters allows us to create a range of input-output transfer functions including linear, sigmoidal and step-like (Figure 1a). Moreover, this transfer function is biologically relevant: protein-protein interactions and enzymatic reactions can be described by Hill function formulations to a good approximation [42], .

In some cases, use of a normalized function is too restrictive for practical application. For example, if model inputs are purely binary (values of either zero or one), the output of a normalized function would also be zero or one, making it impossible for a cFL gate to achieve intermediate states of activation. Accordingly, our cFL method allows for alternative transfer functions. For example, although the method is not limited to binary model inputs, the ligand inputs of our current work are binary (either present or not). If we used normalized transfer functions to relate these model inputs to downstream outputs, all model species would also be either zero or one. Thus, for these transfer functions, we used a constant multiplied by the binary ligand input value (see Materials & Methods).

If more than one input node influences an output node, this relationship is categorized as either an “AND” or “OR” interaction. An AND gate is used when both input nodes must be active to activate the output node, whereas an OR gate is used when either input node must be active. Mathematically, we represent AND behavior by evaluating each input-output transfer function and selecting the minimal possible output node value (*i.e.*, applying the “min” operator, Figure 1c) whereas we select the maximal value (“max” operator; Figure 1d) to evaluate an OR gate. Finally, if both AND and OR gates are used to relate input nodes to an output node, our formalism evaluates all AND gates prior to OR gates. This order of operations corresponds to the disjunctive normal or sum of products form [45].

### Use of cFL to understand experimental data in the context of a prior knowledge network: CellNOpt-cFL

**Figure 2:**
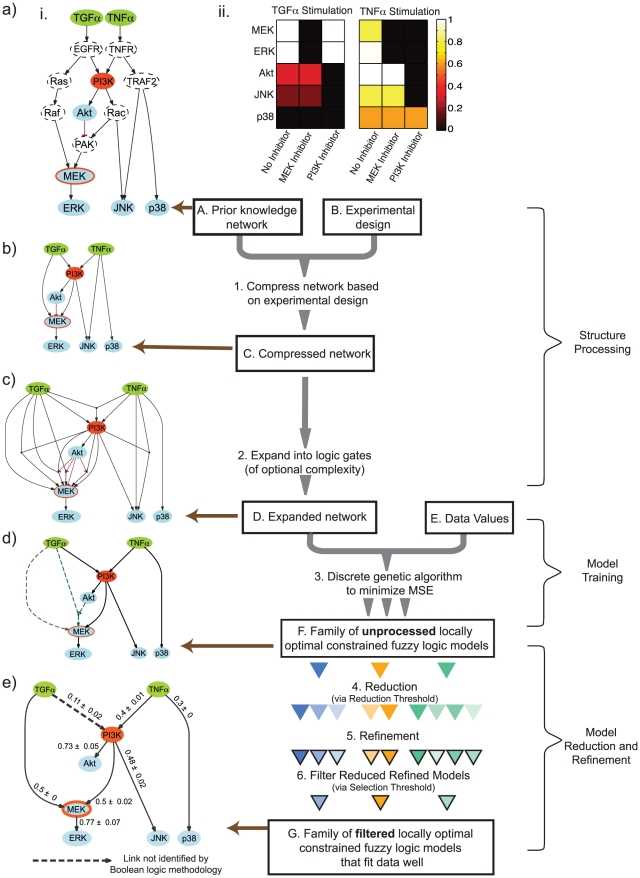
CellNOpt–cFL workflow and application to toy model. *Right side: Workflow (Boxes A through G and Steps 1–6).* The methodology requires a dataset that describes some species in the prior knowledge network (PKN; Box A). Based on the experimental design of the dataset (Box B), the map is compressed to contain only nodes measured (blue nodes), perturbed (green stimulated nodes and orange inhibited nodes), or necessary to maintain logical consistency between nodes (Step 1). The resultant compressed network (Box C) is then expanded to contain multiple possible logic descriptions of gates connecting more than one input node to a single output node (Step 2). The resultant expanded network (Box D) is trained to the data values (Box E) using several independent runs of a discrete genetic algorithm to minimize MSE (Step 3). Each independent run results in an *unprocessed* cFL model represented with a grey triangle. This results in a family of *unprocessed* cFL models (Box F). The result of each independent optimization run is now represented with a different colored triangle. Each individual *unprocessed* model is reduced with several reduction thresholds (Step 4), resulting in several *reduced* models (different triangles shadings). The parameters of each reduced model are then refined (Step 5), resulting in *reduced-refined* models (triangles outlined in black). Finally, one model is chosen to represent each original *unprocessed* model using a selection threshold (Step 6), resulting in a family of *filtered* models (Box G). *Left side: Application to a toy model (panels a to e).* A PKN was hypothesized from the Ingenuity Systems database (www.ingenuity.com) (a.i.) and compared to an *in silico* dataset generated by a simulation of a cFL model with known topology and parameters (a.ii.). The PKN contains 15 molecular species represented as nodes that are believed to positively (arrows) or negatively (blunt arrows) affect others species. These intermediate nodes summarize the possible paths between experimentally stimulated ligands (green) and measured (blue) or inhibited (orange) species. The model was compressed (b) as described in [29] and then expanded (c) to contain all possible two-input AND gates. The expanded network was trained to the *in silico* dataset with twenty independent runs of the discrete genetic algorithm. The topologies of the resultant models (d) were identical except in the case of the gate describing activation of MEK, with sixteen models modeling this interaction with an activating gate (brown, dashed gate) and four models using an AND-NOT gate (green, dashed gate). The TNFα → JNK cFL gate was removed from all *unprocessed* models, reflecting that this interaction was inconsistent with the *in silico* data. The reduction process (Figure 3) showed that the AND-NOT gate could be described more simply without significantly affecting the MSE, resulting in a family of *filtered* models (e). We have labeled each gate with the sensitivity of the gate (defined in Materials & Methods), where sensitivity is scaled between zero and one and a higher sensitivity indicates that the output node is more active at lower input node values. All maps and the graphs of cFL models were generated by a CellNOpt routine using the graphviz visualization engine (www.graphviz.org) followed by manual annotation in Adobe Illustrator.

The process of training a cFL network (CellNOpt-cFL) has two starting requirements. The first is a prior knowledge network (‘PKN’; Figure 2, box A). A PKN depicts interactions among the nodes as a signed, directed graph (such as a PSN) and can be obtained directly from the literature. Alternatively, a large number of commercial (*e.g.*, Ingenuity Systems: www.ingenuity.com; GeneGo: www.genego.com) or academic (*e.g.*, Pathway Commons: www.pathwaycommons.org, reviewed in [46]) pathway databases as well as integrative tools (*e.g.* [47], [48]) can be utilized to construct a PKN. The second requirement is a dataset describing experimental measurements characterizing node activities following stimulation of and/or perturbations in upstream nodes (ligand and inhibitor treatment in our example; Figure 2, box B). CellNOpt-cFL is then used to systematically and quantitatively compare the hypothesized PKN to the experimental dataset.

In practice, available experimental data is usually insufficient to fully constrain both the parameters and topology of the cFL models, and CellNOpt-cFL recovers many models that describe the data equally well. Due to this typical absence of firm structural and parametric identifiability [29], [49], [50], we examine families of models that fit the data equally well rather than attempting to identify a single global best fit. Specifically, we examine interactions in the PKN that were either retained or consistently removed by training. We also use individual models to predict input-output characteristics. This treatment allows us to calculate both an average prediction as well as a standard deviation, which we show below can be useful for discrediting inaccurate predictions.

Our method comprises three main stages (Figure 2): first, structure processing converts a PKN into a cFL model; second, model training trains the model to experimental data; and third, model reduction and refinement simplifies trained models. To illustrate CellNOpt-cFL, we examine a simple toy problem of training a PKN of the phospho-protein signaling network response to TGFα and TNFα (Figure 2a.i) to *in silico* data of activation of several downstream kinases in response to these ligands in the presence or absence of PI3K or MEK inhibition (Figure 2a.ii).

### PKN processing

In the first step, we streamline the network to contain only measured and perturbed nodes as well as any other nodes necessary to preserve logical consistency between those that were measured or perturbed ([29]; Figure 2, Step 1), resulting in a *compressed* PKN (Figure 2 box C). In our example, many nodes that were in the original PKN were neither measured nor perturbed experimentally. Because these nodes could be removed without causing logical inconsistencies, they were not explicitly included in the compressed network (Figure 2b).

In the second step, we expand the network into the multiple logical relationships (combinations of AND and OR gates) that can relate output nodes to their input nodes (Figure 2, Step 2). For example, our toy PKN was expanded to include all possible two-input AND gates governing the response of nodes with more than one possible input node (Figure 2c).

### Model training

In the third step, we train the cFL models to the data (Figure 2, Step 3). We start by limiting the possible parameter combinations to a subset of discrete parameter values that specify seven allowed transfer functions as well as the possibility that the input does not affect the output node (*i.e.* the cFL gate is not present). A discrete genetic algorithm determines transfer functions and a network topology that fit the data well by minimizing the mean squared error (MSE, defined in Materials & Methods) with respect to the experimental data.

Due to the stochastic nature of genetic algorithms, multiple optimization runs return models with slightly different topologies and transfer function parameters that result in a range of MSEs. Models with an MSE significantly higher than the best models are simply eliminated from further consideration. Models with similar MSEs but different topology and parameters result from the insufficiency of the data to constrain the model such that each model fits the data well albeit with slightly different features. We consider each individual in this group as a viable model, and all are included for subsequent analysis. Thus, after multiple independent optimization runs using the discrete genetic algorithm to train the expanded PKN against the data, a family of models with transfer functions chosen from a discrete number of possibilities is obtained.

For each of these models, we generate *unprocessed* models (Figure 2, box F) by removing all cFL gates that are logically redundant with other cFL gates (*e.g.*, in the gate “(B AND C) OR B activate D”, the AND gate is logically redundant with the “B activates D” gate). These gates are removed because they increase model complexity by using multiple logic gates to encode a relationship that can be specified by a simpler gate.

In our toy example, a family of twenty *unprocessed* models was obtained by training the expanded map (Figure 2c) to *in silico* data (Figure 2a.ii.) using the discrete genetic algorithm. The *unprocessed* models from different optimization runs had similar topologies with the exception of the gate describing the relationship of MEK to its input nodes: TGFα and Akt (Figure 2d, brown and green dashed gates). Sixteen of the *unprocessed* models described the activation of MEK as depending only on TGFα (brown, dashed gate) whereas four described activation using the AND NOT gate (green, dashed gate).

### Model reduction and refinement

**Figure 3:**
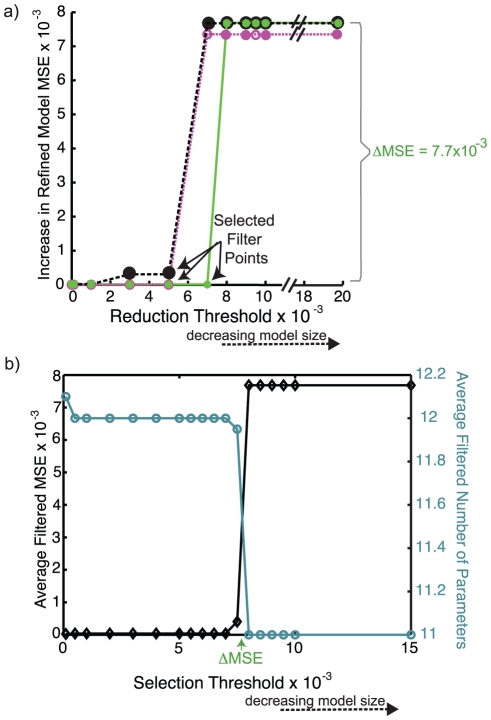
Reduction of trained cFL models. The *unprocessed* models resulting from twenty independent runs of the discrete genetic algorithm to train the expanded network to an *in silico* dataset were reduced using several reductions thresholds and subsequently refined. The behavior of three representative models is shown (a). To develop a criterion for our model selection, we note that each individual model exhibits a drastic increase in refined MSE when reduced at some reduction threshold. For our toy model, the MSEs of some *reduced-refined* models increase significantly (ΔMSE of 7.7x10^−3^) at a reduction threshold of greater than 5×10^−3^ (a., magenta line), whereas the MSEs of others only increase at a reduction threshold greater than 7×10^−3^ (a., green line). This increase in MSE of 7.7×10^−3^ is deemed significant because it corresponds to the models no longer fitting the *in silico* data of Akt and JNK under TGFα stimulation (the remaining data are still well fit). For each *unprocessed* model, we refer to the reduction threshold above which a significant increase in MSE is observed as the ‘filter point’ of the model. Each individual model has a filter point that is determined based on the amount that the *reduced-refined* model's MSE is allowed to increase. We term this allowable increase in MSE the ‘selection threshold’. For example, one model of our toy example (black line) could be described as having a filter point of 1×10^−3^ or 5×10^−3^, depending on the amount of increase in MSE allowed by the selection threshold. To choose a selection threshold, we compare the average increase in final MSE to the average decrease in the number of parameters in the resultant *filtered* family of models (b) and note that, at a selection threshold of 7.7×10^−3^, the average MSE increases while at a selection threshold of 5×10^−4^, average number of parameters decreases. Thus, a selection threshold of 5×10^−4^ to 7.6×10^−3^ results in the models at the “filter points” noted in (a).

In the model reduction and refinement stage (Steps 4–6), we determine which gates can be removed altogether as well as AND gates that can be replaced with one-input cFL gates without significantly affecting the MSE. We implemented the non-exhaustive heuristic search procedure described below on each *unprocessed* model and illustrate its application to our toy example (Figure 3).

In the fourth step, we remove or replace all gates for which the alteration does not increase the MSE of the *unprocessed* model over some threshold, which we term the ‘reduction threshold’. We use a range of reduction thresholds such that each *unprocessed* model results in several models, one for each reduction threshold used. Following this step, the resultant models are considered *reduced* models.

In the fifth step, we fix the model topology to that obtained during Step 4 and treat the transfer function parameters in each *reduced* model (Figure 2, Step 5) as continuous parameters rather than the discrete set of transfer function parameters required for use of the discrete genetic algorithm. We use a Sequential Quadratic Programming method (Text S1) to refine the model parameters and further improve the fit of the models to the experimental data. The resulting models are termed *reduced-refined* models, which have a range of MSEs depending on the reduction threshold used (Figure 3a).

In the sixth and final step, we specify a *reduced-refined* model to represent each *unprocessed* model (Figure 2, Step 6). For each *unprocessed* model, we choose the *reduced-refined* model that has the fewest number of fitted transfer function parameters without increasing the MSE above a defined ‘selection threshold.’ The selection threshold is chosen by comparing the average number of parameters in the family of models to the average MSE of the models (Figure 3b). The net result is a set of *reduced-refined-filtered* models (hereafter referred to as *filtered* models, Figure 2, Box G).

In our toy example, the *filtered* models have identical topology and in no case does Akt inhibit MEK activation (Figure 2e). This topology is, in fact, the topology from which the *in silico* data was derived. The ability of cFL to fit intermediate values made it possible to recover the correct model topology, whereas BL did not identify the correct model, and a gate linking TGFα to PI3K was consistently missing (Figure 2e, dashed arrow). Specifically, BL was unable to return the correct topology because nodes downstream of PI3K (Akt and JNK) were partially activated (0.32 and 0.19, respectively) under conditions of TGFα stimulation, and a BL model that included the TGFα to PI3K gate had a higher error (MSE  = 0.56) than a model that omitted the interaction (MSE  = 0.07). In contrast, the improved ability of cFL to model graded activities made it possible to recover the true network topology.

### Adjusting the complexity of CellNOpt-cFL model training

While the expansion step (Figure 2, step 2) captures the many possible combinations of AND and OR logic relationships between nodes, it also increases the complexity of the network, resulting in an increase in the size of the optimization problem. Depending on the biological network of interest, some or most of these AND gates might not be biologically relevant. For example, it is unlikely that six receptors must be active in order to activate another species, as would be the case for a six-input AND gate (instead, it is more likely to be a OR gate). A profusion of AND gates also makes the resultant networks difficult to interpret because most AND gates are in only a few models whereas the majority of models contain single-input and OR gates. Thus, the AND gates can effectively appear as system “noise”, interfering with visual assessment as well as computational analysis of the model topologies. Because of these potential complications, the expansion step can be limited to include only AND gates with a few inputs, depending on the complexity one would like to capture with the trained network models.

In the current paper, we have limited the search in the discrete genetic algorithm to a set of seven transfer functions. Use of more or fewer transfer functions is possible, but we found that seven transfer functions allowed us to represent a variety of input-output relationships without unduly increasing problem complexity to the point that the discrete genetic algorithm no longer consistently returned models that fit the data well (see Materials & Methods).

### Applying CellNOpt-cFL to protein signaling data from HepG2 cells

**Figure 4:**
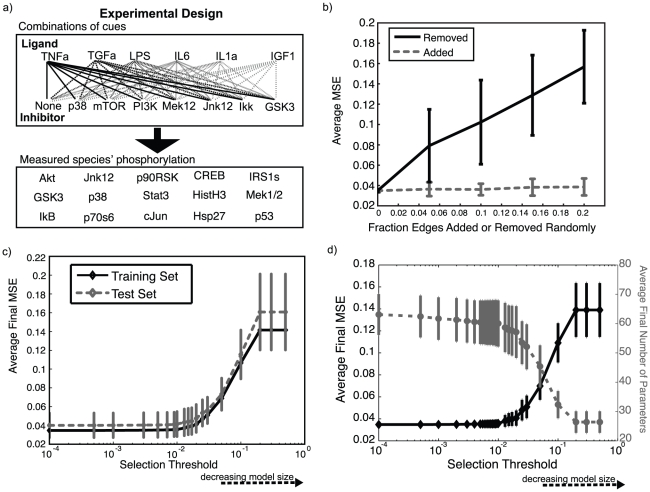
Initial analysis of cFL models trained to HepG2 dataset. (a) Experimental design of a dataset describing the measured signaling response of the HepG2 cell line to six ligand stimulations in the presence or absence of inhibition of seven species. CellNOpt-cFL was used to train the PKNs (Figure S2) to this dataet. (b) The fraction of edges indicated were randomly removed from (solid line) or added to (dashed line) PKN1^i^ to result in at least 90 altered PKNs, which were subsequently trained to the HepG2 data. The average MSEs of the altered PKNs indicates that removal of edges reduced the ability of the trained models to fit the data (solid line). Because CellNOpt-cFL does not add links to the model, this result is as expected. The addition of edges to the PKN did not reduce the ability of the trained models to fit the data (dashed line) since edges that were inconsistent with the data could be removed during the training process (Figure S6). (c) Results of ten-fold cross-validation in which the data was randomly divided into ten subsets and the optimization procedure performed to obtain a family of at least 57 models from training data comprising nine of the ten subsets; the remaining subset was considered a test set. We thus obtained ten families of trained models, one family from the use of each subset as a test set. The fit of these families of models to their respective training and test sets was then plotted as a function of the selection threshold. As expected, on average the ability of the trained models to fit the test sets was slightly worse than, but comparable to, the ability to fit the training sets, suggesting that the models were predictive. The difference between MSEs of the test versus training sets did not change as a function of the selection threshold, suggesting that the models were not overfit, even at very low selection thresholds. (d) A comparison of the average final MSE with the average final number of parameters was used to determine a range of selection thresholds (1×10^−3^ – 1×10^−2^) where the family of models has a slightly lower average number of parameters without greatly increasing the MSE.

To test the ability of cFL modeling to analyze real biological data, we modeled a set of measurements describing the response of the HepG2 hepatocellular carcinoma cell line to various pro-survival, pro-death, or inflammatory cytokines in the presence or absence of specific small molecule kinase inhibitors. This dataset was used to construct a recent BL model [29]. Here we ran an independent analysis using the cFL approach and compare the results to the BL previously reported. The dataset comprises measurement of phosphorylation states as markers of activation of 15 intracellular proteins before and 30 minutes after stimulation by one of six cytokines in the presence or absence of seven specific small molecule kinase inhibitors (Figure 4a, Figure S1). The measurements were normalized to continuous values between zero and one using a routine implemented in the MATLAB toolbox DataRail [51], as previously described ([29], see Text S1).

The HepG2 dataset was trained to several related PKNs which are enumerated in Table 1 and Figure S2. These PKNs were derived, with various extensions, from the Ingenuity Systems database (www.ingenuity.com) with manual addition of literature data about IRS1 that was obviously missing [29]. The first PKN, termed PKN0 was identical the one used previously for BL modeling [29]. In the course of our analysis, we found it necessary to search the literature for interactions missing in PKN0 but supported by the data, resulting in several PKNs (Table 1). Furthermore, we limited the manner in which the PKNs were expanded in two ways: (1) expansion into all possible two-input AND gates or (2) expansion into a two-input AND gate only when one input was inhibitory. In the second case, the expansion of inhibitory gates was necessary because, in logic terms, an inhibitory gate indicates that the output node is active when the input node is not active. In biological networks, this is true if the output node is constitutively active, which was not observed in the normalized HepG2 data. Thus, in order to accurately model the inhibitory effect, it had to occur in conjunction with activation by some other input node, which is captured by an AND gate. If a PKN was processed with both types of expansion, we include a superscript to differentiate between the two cases – *i.e.*, PKN1^a^ for the expansion of all gates and PKN1^i^ for the expansion of only the inhibitory case.

**Table 1:** Prior knowledge networks trained to HepG2 dataset.

	PKN0	PKN1		PKN2				PKN3
Model ID	PKN0	PKN1^a^	PKN1^i^	A	B	C	D	PKN3
**Model Basis**	Ingenuity Database							
**ERK → IRS1 ** [**53**]		X	X	X	X	X	X	X
**TRAF6 → MEK ** [**52**]		X	X	X	X	X	X	X
**Assay → PI3K**				X	X	X	X	X
**IL6R →PI3K ** [**54**]		X	X		X	X		
**IL6R → Ras ** [**54**]						X	X	X
**Protein Signals → Cytokine Release**								X
**Gates expanded into all possible 2-input AND gates (Step 2)**	All	All	Only Inhibitory	Only Inhib.	Only Inhib.	Only Inhib.	Only Inhib	Only Inhib.

PKN0: Initial PKN shown to be insufficient for fitting HepG2 data.

PKN1: Extended PKN used to compare two expansion limitations; PKN1^i^ was used for the majority of subsequent analysis.

PKN2: PKNs used to determine mechanism of IL6-induced protein phosphorylation.

PKN3: PKN further extended to model cytokine release.

### CellNOpt-cFL training of PKN0

PKN0 was expanded to include all possible two-input AND gates and trained to the HepG2 dataset with CellNOpt-cFL (Figure S2). The 90 *unprocessed* cFL models obtained after training showed that PKN0 exhibited a poor fit to IL1α-induced protein phosphorylation (Figure S3), a result we had also observed with BL analysis [29], confirming that the poor fit of BL was due to errors in the topology of PKN0 and not the inability of Boolean logic to fit intermediate values.

An inspection of systematic model/data disparity (Figure S3) immediately indicated that the models did not fit IL1α-induced phosphorylation of IRS1, MEK and several species known to be modulated by the MEK pathway. In PKN0, no paths between IL1α and MEK or IRS1 were present. Based on careful reading of the literature, we added two links to PKN0: a TRAF6 → MEK link [52], and an ERK → IRS1 link [53]. These links had been inferred by the BL framework [29] and were supported by further literature evidence. To add a link that provided a path between IL1α and MEK in the absence of BL inference results, for simplicity one should first consider links from species that IL1α is already known to activate. In this case, TRAF6 is the most upstream species which experimental evidence suggests can activate MEK [52]. In the case of IRS1 signal activation, the specific phosphorylation site measured should be considered. Our data included measurements of phospho-S636/639, and S636 is a known phosphorylation site of ERK2 [53].

A novel finding from CellNOpt-cFL analysis of the HepG2 data was that IL6 treatment led to phosphorylation of several downstream proteins. Similarly to the links just considered, PKN0 included no paths between IL6 stimulation and these downstream proteins, resulting in an inability to fit this pattern of phosphorylation. Importantly, however, BL analysis would not have recognized this partial activation due to its inability to fit intermediate values (as illustrated in our earlier toy example). Because IL6 was observed to partially activate Akt in the data and known mechanisms exist for this activation [54], we added a prospective IL6R → PI3K link to the PKN, thus providing an extended PKN (PKN1) that we use below for subsequent CellNOpt-cFL analysis.

### CellNOpt-cFL training of PKN1

PKN1 was expanded to include all possible two-input AND gates (PKN1^a^) for a total of 170 discrete parameters corresponding to 105 logic gates. The resultant network was trained to the HepG2 data. Reduction of the PKN1^a^–derived models indicated that almost all AND gates could be removed or replaced by single-input gates. Since the AND gates appeared to add unnecessary complexity to the cFL models, we also expanded PKN1 to only include AND gates if an input node was inhibitory (PKN1^i^; Table 1), resulting in only 60 discrete parameters corresponding to 56 logic gates. We then compared the PKN1^a^- and PKN1^i^-derived cFL models.

The comparison of these two PKN-derived model families revealed a clear tradeoff between model fit and complexity. The more complex PKN1^a^-derived models were able to fit the data slightly better than the PKN1^i^-derived models (average *unprocessed* model MSE of 0.032±0.002 compared to 0.035±0.002, *p*<0.001). However, the more complex PKN1^a^-derived models contained many more parameters than the PKN1^i^-derived models both before and after optimization (170 compared to 60 discrete parameters before optimization and an average of 72.8±4.9 compared to 66.6±3.9 continuous parameters after optimization (*p*<0.001); Figure S4). The simpler PKN1^i^-derived models used fewer initial and final parameters to arrive at a fit to the data only 9% worse than PKN1^a^-derived models. Since the 9% deviation is in the range of error in the normalized data (error estimated to be 10% by comparing similar stimulation conditions), we focused subsequent analysis on the simpler PKN1^i^-derived models. For completeness, we include the results of PKN1^a^-derived models as supplemental information (Figure S5).

### Statistical significance of cFL models trained to PKN1^i^

To determine the statistical significance of our results, we compared the family of 243 *unprocessed* models with *unprocessed* models obtained from either training PKN1^i^ to randomized data or training a randomized PKN1^i^ to the data (Table S1). Data was randomized by pairwise exchange of all data values while network topologies were randomized either by generation of an entirely random topology or by random pairwise exchange of gate inputs, gate outputs, or nodes' inputs [29]. When compared to the results of all types of randomization, models trained to the real data and PKN1 were highly significant (P-value <0.001, Table S1), indicating that the family of trained cFL models fit the data better than expected by random chance.

To probe the dependence of the CellNOpt-cFL training process on the quality of the PKN used, we randomly added links to or removed links from the PKN and trained the resultant PKN to the data. As expected, the models derived from PKNs with links randomly removed had a poorer fit to data than those derived from the complete PKN1^i^ (Figure 4b, solid line). Conversely, when links were randomly added to the PKN, cFL-CellNOpt effectively removed the links (Figure S6), resulting in models with similar goodness of fit as models derived from PKN1^i^ (Figure 4b, dashed line). We thus conclude that an incomplete PKN degrades the ability of CellNOpt-cFL to fit the data whereas models derived from a PKN with extraneous links retain this ability.

As an initial investigation of model predictive capacity and a check for over-fitting, we performed a ten-fold cross-validation by randomly dividing the HepG2 data into ten subsets and, for each subset, reserving one as a test set while training with the remaining nine data subsets. The similar fits of the training and test data provided evidence that the family of models obtained from this procedure were predictive, and the difference in test and training MSEs did not depend on selection threshold, a measure of model size, suggesting that the models were not over-fit (Figure 4c).

Analysis of this cross-validation result combined with a plot of average *filtered* model size and fit (MSE) as a function of selection threshold (Figure 4d) suggested that a selection threshold in the range 1×10^−3^ – 1×10^−2^ would result in a family of models that contain slightly fewer number of parameters than lower thresholds (Figure 4d, dashed line) while retaining the ability to fit the data well (Figure 4d, solid line). We used a threshold of 5.0×10^−3^ for the remainder of our analysis unless otherwise noted.

**Figure 5:**
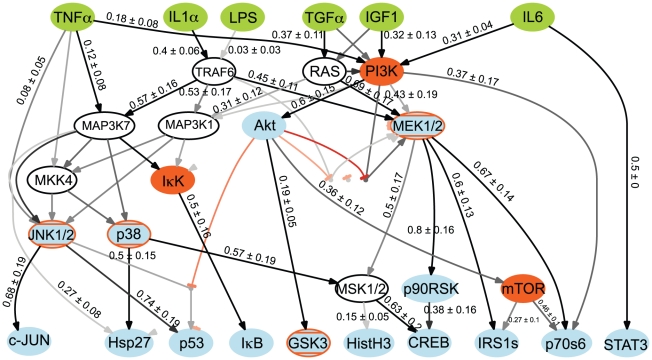
Structure of family of cFL models resulting from training PKN1^i^ to HepG2 dataset. Topologies of the family of *filtered* cFL models trained to the HepG2 dataset. *Unprocessed* cFL models can be found in Figure S6 and fit of the *filtered* models to the data in Figure S7. Nodes represent proteins that were either ligand stimulations (green), inhibited (orange), measured by a phospho-specific bead-based antibody assay (blue), or could not be removed without introducing potential logical inconsistency (white). The grey/black intensity scale of the gates corresponds to the proportion of individual models within the family that include that gate. Thus, links colored black were present in all models whereas links colored grey were present in a fraction of the models. Where visually feasible, cFL gates are labeled with a numerical value that corresponds to a quantitative sensitivity of the input-output relationship. Sensitivity is calculated as described in the Materials & Methods. The larger this value, the lower the level of the input nodes' activity required for generating significant output node activity (*i.e.* a gate with a high sensitivity indicates that the output node is sensitive to a low value of its input node). The uncertainties in these values arise from the various best-fit EC_50_ for each individual model. The graph of the cFL models was generated by a CellNOpt routine using the graphviz visualization engine (www.graphviz.org) followed by manual annotation in Adobe Illustrator.

Finally, we obtain a family of 243 *filtered* models for further analysis (Figure 5). By taking note of which cFL gates are removed during the CellNOpt-cFL training and reduction processes, one can generate hypotheses regarding these gates. Table 2 summarizes a set of biological hypotheses readily suggested by our cFL model topologies.

**Table 2:** Biological hypotheses about signaling network operation suggested by gates removed during CellNOpt-cFL analysis.

Hypothesis	Evidence in cFL Models	Evidence in data
Akt → Iκk crosstalk is inconsistent with the data.	Akt → Iκk gate is not present in *unprocessed* models (Figure S6)	Phosphorylation of Akt and Iκb are not positively correlated (correlation coefficient of −0.24).
Crosstalk from the growth and survival pathways (MEK/ERK and PI3K/Akt) to the inflammatory pathways (Nfκb, JNK, and p38) is not necessary to fit the data well.	Akt → Iκk gate is not present in *unprocessed* models and frequencies of other relevant crosstalk gates (Ras → MAP3K1 and PI3K → MAP3K1) are low in *unprocessed* models and decrease in *filtered* models.	
Crosstalk from the MEK/ERK pathway is not necessary to describe Hsp27 phosphorylation.	MEK → Hsp27 gate is not present in *unprocessed* models.	Phosphorylation of MEK and Hsp27 is not strongly correlated (correlation coefficient of 0.43) but phosphorylation of JNK and Hsp27 is strongly correlated (correlation coefficient of 0.91)
HistH3 data is not well described by PKN1.	Frequency of MSK1/2 → HistH3 gate is low in *unprocessed* models and decreases in *filtered* models and models do not fit HistH3 data well (Figure S7)	Phosphorylation of HistH3 and neither MEK nor p38 are strongly correlated (correlation coefficients of 0.55 and 0.47, respectively)
LPS does not activate the measured signaling nodes.	Frequency of LPS → TRAF6 gate is low in *unprocessed* models and decreases in *filtered* models.	The only protein that is consistently phosphorylated under LPS stimulation is Akt

### Validated biological hypothesis 1: Crosstalk from TGFα to the JNK pathway

Analysis of error between the family of cFL models and experimental data (Figure S7) highlighted consistent error in TGFα-induced partial activation of c-Jun. Both PKN0 and PKN1 allowed for TGFα-induced activation of c-Jun by the JNK pathway via crosstalk from Ras or PI3K to MAP3K1. In the BL methodology, this crosstalk was removed due to the inability to fit partial activation, and no BL model allowed for activation of c-Jun after TGFα stimulation. However, we found that a subset of cFL models accounted for this c-Jun partial activation by including crosstalk between Ras or PI3K and MAP3K1. These models also partially activated JNK after TGFα stimulation, a feature that was inconsistent with the training data (Figure S8). Thus, these models predict that JNK was actually phosphorylated under conditions of TGFα stimulation, but our measurements did not detect it.

**Figure 6:**
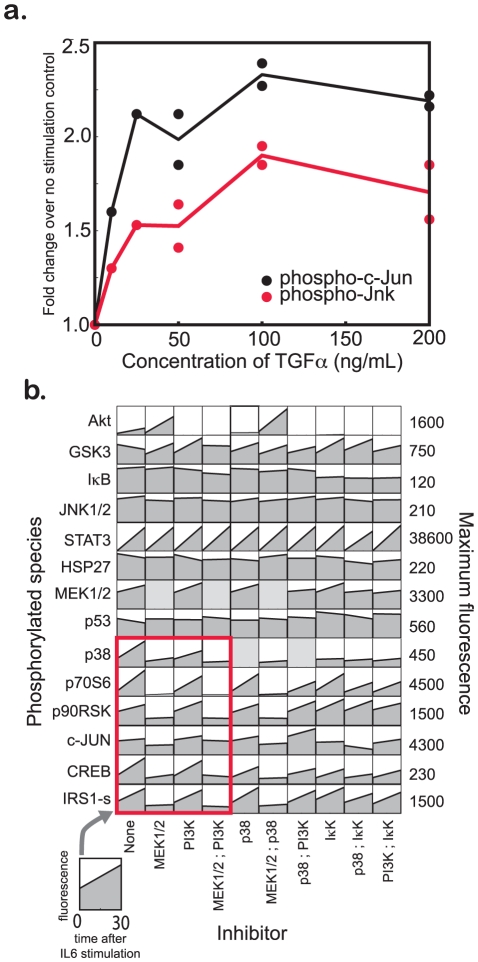
Validation of cFL crosstalk predictions. (a) Analysis of systematic error as well as the topologies of the family of trained cFL models (Figure 5) indicated that c-Jun was partially activated after TGFα stimulation. Models with crosstalk from Ras or PI3K to Map3K1 predicted that JNK was partially activated under these experimental conditions even though it was not partially activated in the dataset. We tested whether JNK was actually partially activated under these conditions by stimulating HepG2 cells with TGFα and measuring levels of phosphorylated JNK and c-Jun by a bead-based antibody assay after 30 minutes. Fold increase in measured phosphorylation over un-stimulated control for c-Jun (black) and JNK (red) is shown. Where available, biological replicates are indicated with filled circles. Solid lines indicate the averages of the replicates. This experiment indicates that JNK was partially phosphorylated under TGFα stimulation and the cFL models with crosstalk from Ras or PI3K to MAP3K1 were correct. (b) CFL analysis of the topologies and fit of the HepG2 training dataset to several PKNs suggested that IL6 activated downstream nodes through the Ras/MEK pathway (Table 3). To test this prediction, a validation dataset was examined [36]. This validation dataset showed that the activation of nodes other than STAT3 that responded robustly to IL6 stimulation was ablated by pretreatment with a small molecule MEK inhibitor but not other inhibitors, demonstrating that the Ras/Raf/MEK pathway mediates this crosstalk.

To test this prediction directly, we undertook *de novo* measurement of JNK and c-Jun phosphorylation following stimulation with different doses of TGFα (Figure 6a). These new data show that JNK does indeed become phosphorylated upon stimulation of HepG2 cells with TGFα. Thus, the cFL models containing crosstalk from Ras or PI3K to MAP3K1 were the correct models. Combined with Table 2, this analysis highlighted the partial activation of the JNK pathway after TGFα stimulation as a singular instance of crosstalk from a pro-growth ligand to an inflammatory pathway. In support of the significance of our finding here, we note that TGFα-induced JNK activation has been shown to be important for hepatic regeneration [55] and stimulation of DNA synthesis [56] in primary rat hepatocytes.

### Validated biological hypothesis 2: Mechanism of IL6-induced protein phosphorylation

As previously mentioned, PKN0 was unable to fit IL6-induced protein phosphorylation (a feature of the data unappreciated by the BL methodology). Because Akt was observed to be partially phosphorylated under these conditions and we found literature evidence for a prospective IL6R → PI3K link, we added the link to PKN1. However, the media-only condition also induced partial phosphorylation of Akt. Discovery of the partial activation of Akt in the media-only control led us to consider that perhaps the IL6-induced phosphorylation of Akt was simply an assay artifact. Thus, we inserted an Assay → PI3K link into the PKN. This “Assay” node represents cell stress arising from changing environmental conditions during the assay (media change, *etc.*); it is postulated to activate PI3K because only Akt is consistently active in the untreated control. Having accounted for the potential that IL6-induced partial phosphorylation of Akt was an artifact, we undertook a series of computational experiments to determine the mechanism of IL6-induced phosphorylation of downstream proteins.

Upon exposure to IL6, SHP2 has been reported to bind to gp130, a subunit of the IL6 receptor complex. SHP2 is then phosphorylated in a JAK1-dependent manner. This phosphorylation can lead to PI3K/Akt pathway activation through interactions with Gab-1 or IRS1 or Ras/MEK/ERK pathway activation through Grb2 or Gab1 [54]. Thus, our computational experiments were designed to infer which pathway (PI3K/Akt or Ras/MEK/ERK) was mediating the IL6-induced protein phosphorylation. Four families of 150 *filtered* models were examined, all of which were obtained after training a new PKN to the normalized HepG2 dataset (Table 3, PKN2A – PKN2D). The inability of PKN2A-derived cFL models with only the Assay → PI3K link to fit well the IL6-induced protein phosphorylation data suggested that some other link was necessary to fit this data. In our trained networks, the IL6R → PI3K link was present in only a fraction of the relevant trained models (PKN2B and PKN2C), but the IL6R → Ras link was present in more than 90% of relevant trained models (PKN2C and PKN2D). Additionally, models with IL6R → Ras links were better able to fit the IL6-induced protein phosphorylation. Consequently, our cFL results supported the hypothesis that IL6R activates downstream proteins through the Ras/Raf pathway. This hypothesis is supported by an independent dataset [29], where the IL6-induced protein phosphorylation response was more robust than in the training data (Figures S1 and S9). Inhibition of MEK either alone or in combination with other inhibitors resulted in ablation of downstream protein activation whereas inhibition of PI3K did not (Figure 6b). Thus, we infer that IL6-induced protein phosphorylation was not an assay artifact and was instead mediated by the Ras/Raf pathway.

**Table 3:** Results of cFL training of various prior knowledge networks for the investigation of IL6 crosstalk.

PKN	Assay to PI3K?	IL6R to PI3K?	IL6R to Ras?	MSE_IL6_
PKN1^i^	-	100%	-	0.040±0.004
PKN2A	100%	-	-	0.052±0.004
PKN2B	97%	56%	-	0.046±0.008
PKN2C	99%	40%	95%	0.028±0.004
PKN2D	99%	-	98%	0.028±0.004

### Predicting node-to-node transfer functions

**Figure 7:**
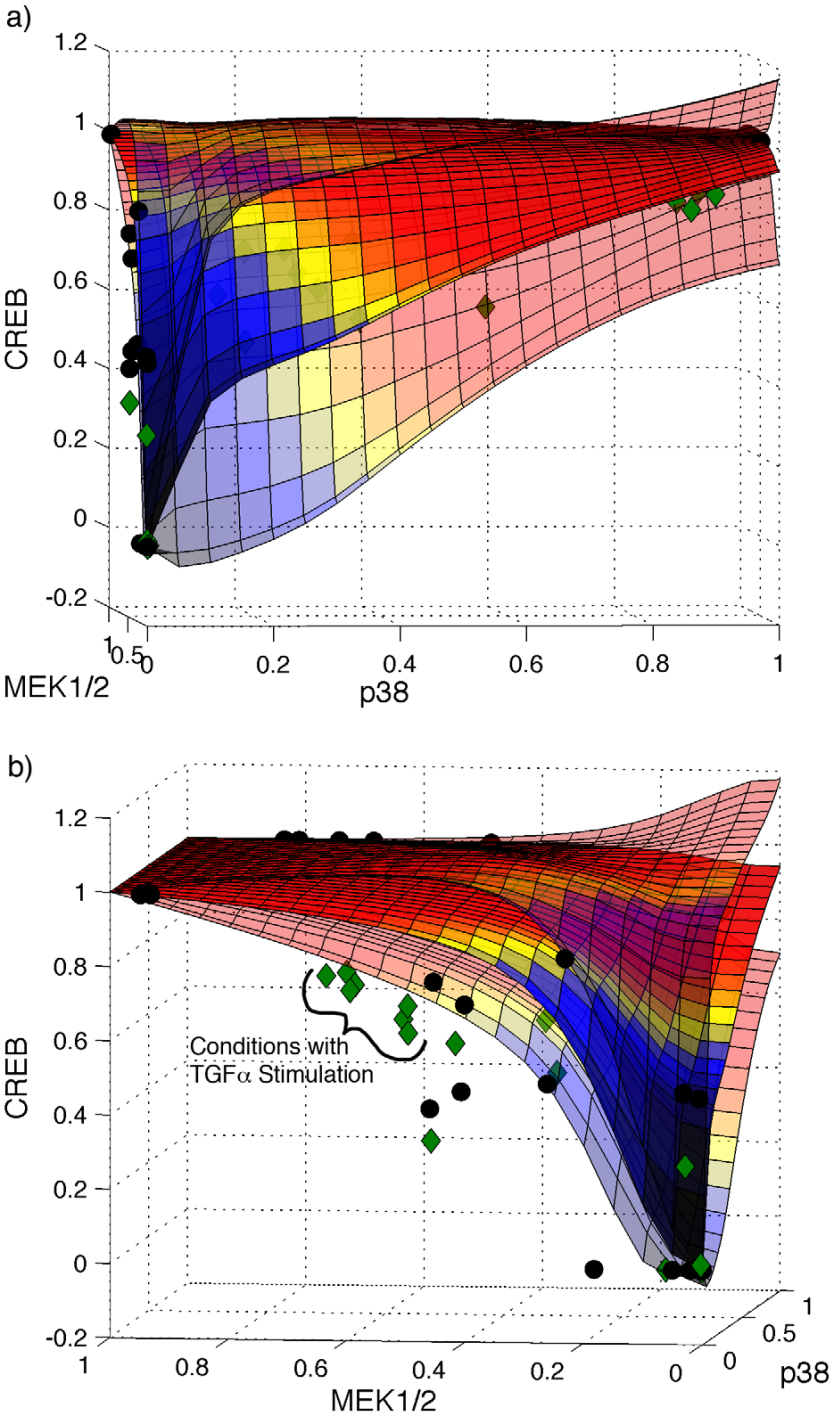
Transfer functions predicted by trained cFL models. The output value of the CREB node was predicted by computationally simulating each individual model in the family of cFL models with 441 combinations of p38 and MEK1/2. Three-dimensional plots were generated in MATLAB showing the average prediction (opaque surface) as well as the average prediction plus or minus the standard deviation of the predicted value (semi-transparent surfaces). The training data (black circles) and validation data (green diamonds) are also plotted. The 3-D plots have been rotated to highlight the influence of either (a) p38 or (b) MEK1/2. The predicted transfer functions agree with the validation data reasonably well except for the overestimation of CREB activation for conditions with TGFα stimulation as one of the ligands.

CFL relates nodes in a network with transfer functions that describe quantitative input-output relationships between protein species represented as network nodes. To investigate the ability of the cFL models to predict these transfer functions, we simulated the PKN1^i^-derived, *filtered* cFL models to determine the activation state of a specified node under many theoretical combinations of its input nodes. We then plotted the model predictions of quantitative input-output relationships. As one instance, Figure 7 shows the predicted average and standard deviation of the quantitative values of CREB phosphorylation as a function of the activation of upstream nodes, p38 and MEK1/2. The resulting plots indicated that we were able to predict the activation response of CREB to the entire range of p38 and MEK1/2 although training set measurements were limited to a few values of these nodes (Figure 7, black circles).

We tested this prediction using a set of data with combinations of ligands and inhibitors not present in the training data ([29], Figure S9). Roughly 20% of the test conditions were also present in the training data set, allowing us to control for differences between both data sets. When we compared this dataset to the predicted transfer functions, we observed that most of the data fell within one standard deviation of the predicted value (Figure 7, green diamonds) with exception of overestimation under conditions of TGFα stimulation. This overestimation is expected, as a comparison of common conditions between the training and test dataset indicated that the normalized experimental values of CREB in the validation dataset were 38±4% lower than that in the training set.

This result demonstrates the ability of the trained cFL models to predict the quantitative relationship between nodes in the network. We also found that the family of cFL models was able to fit the phospho-protein signaling response in the validation dataset well, which we demonstrate as supplementary information (Figure S9).

### Predictive capability of a cFL model family

We performed a series of nineteen cross-validation experiments to further investigate the ability of our methodology to predict the signaling response under conditions that were not represented in the training data. For each experiment, we used training data from which we had removed the phosphorylation data of a specific protein signal, *s*, under a single ligand stimulation condition and all inhibitor treatments. Nineteen signal/stimulation combinations were chosen to be test sets according to two criteria: (1) *s* is at least partially activated under the stimulation condition of interest and (2) *s* is at least partially activated under some other stimulation condition (Table S2). These criteria ensured that the remaining training data contained some information regarding the activation of *s* but it did not contain information regarding the activation of *s* under the stimulation condition of interest. This procedure is a more stringent test for predictive capability than a random cross-validation procedure because training sets from which random data is removed might retain other data with the same information as the removed data (*e.g.*, based on the network topology, Akt phosphorylation in the absence of MEK inhibition is the same as Akt phosphorylation with MEK inhibition, so removing only one of these data points is not a stringent test of predictive capacity).

**Figure 8:**
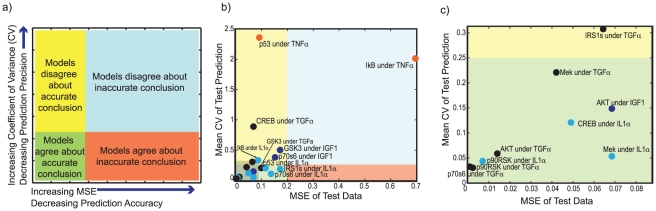
Accuracy vs. precision of cross-validation experiments. (a) Model predictions can be assessed based on both how well the family of models agree on a prediction (precision) as well as their accuracy. If a prediction is imprecise (*i.e.* the models do not agree), the models are not constrained to any single prediction. Thus, precision can be used to discredit predictions. Predictions can be both precise and accurate (green field), imprecise but accurate on average (yellow field), imprecise and inaccurate (blue field), or precise but inaccurate (orange field). Predictions that are precise and accurate (green field) are preferred. (b) The importance of considering the precision of a prediction amongst a family of models was demonstrated by a cross-validation study in which a signal under a single ligand stimulation condition in the presence or absence of any inhibitor was removed from the training data set. The mean coefficient of variance (CV) as a function of the error in the prediction (MSE) is plotted for all tests. One prediction was highly inaccurate. However, it was also imprecise (blue field), whereas no predictions were precise and inaccurate (orange field), demonstrating that taking the precision of a prediction into account can help to discredit inaccurate predictions. (c) The grey-boxed subset of (b) highlights the test sets that were precisely and accurately predicted by the family of cFL models.

We examined the ability of models trained on reduced training sets (n>45 for each case) to predict phosphorylation of the test protein signals. Because we used each individual in the family of models to predict the test signal, we could determine if the models were constrained in their predictions by examining the coefficient of variance (CV; standard deviation divided by mean) of the prediction. If the CV was high, the models were not constrained to a specific prediction (*i.e.* the prediction was imprecise), and the average prediction should be discounted. Thus, for these cross-validation results, we compared the precision (CV) and accuracy (MSE) of the models' predictions, where precise and accurate predictions exhibited both a low CV and low MSE (Figure 8a).

We found that the families of models trained on these reduced training sets were able to precisely predict phosphorylation of the test protein signals in twelve of the nineteen cases (Figure 8b and c, green field). In six of the test sets, the models did not agree, although their average prediction was reasonably accurate (Figure 8b and c, yellow field). We observed no test sets for which the training sets agreed about an inaccurate prediction (Figure 8b, orange field). In one case (prediction of Iκb signaling under TNFα stimulation), the predicted phosphorylation state was highly inaccurate (MSE >0.20). However, this prediction was also very imprecise (CV >0.25), indicating that the average prediction was unreliable (Figure 8b, blue field). Thus, by taking the precision of the models' predictions into account, we were able to discredit an inaccurate prediction. This result underscores the importance of considering consensus among the family of models rather than examining the results of only one cFL model.

### Using cFL models to relate phospho-protein signaling to cell phenotypic response

The ability to quantitatively model protein signal activation with cFL offers the prospect of predicting phenotypic response upon exposure to stimuli and inhibitors. To investigate the ability of cFL to model phenotypic data, we turned to data describing cytokine release three hours after stimulation under the same conditions as the phosphorylation data [36]. As a first approach, we linked the output of our family of cFL models to a partial least squares regression model [6] obtained by regressing normalized data of release of five cytokines (IL1β, IL4, G-CSF, IFNγ, and SDF1α) to the normalized protein phosphorylation measurements (see Text S1).

The cFL models linked to a PLSR model were able to model phenotypic response with an accuracy of R^2^ = 0.79, near that of the PLSR model (R^2^ = 0.81; see Figures S10). However, we found that the correlation indicated by regression coefficients did not lead to easily interpretable insights about phenotype because proteins in the same pathway were also highly correlated with each other.

To obtain a more interpretable model, we utilized a second approach where we included nodes specifying cytokine release in the PKN and linked them to a few protein signaling nodes. These nodes were chosen based on principle component analysis: if protein signals in a pathway clustered together in principle component space, the signal most downstream in the pathway was linked to cytokine release. Based on this analysis, the following protein signaling nodes were linked to each cytokine release node: MEK1/2, CREB, GSK3, c-Jun, Hsp27, Iκb, and STAT3 (Table 1, PKN3). We then trained a family of cFL models to the normalized dataset comprised of cytokine release at three hours and protein signaling at thirty minutes.

**Figure 9:**
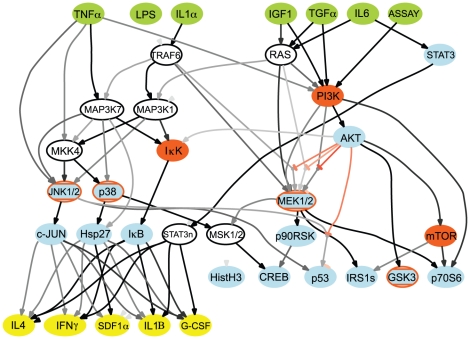
Trained cFL models linking ligand cues, phospho-protein signals, and cytokine release phenotypic responses. A dataset describing release of five cytokines after three hours under conditions identical to those under which protein phosphorylation was measured was combined with the phospho-protein dataset. PKN2D was further extended to include links from protein signals that occupied unique principle component space (Text S1) to nodes of cytokine release after three hours. Training this network to the data indicated that the growth and survival pathways were not needed to describe cytokine release. Thus, the PKN was revised to link only Stat3, NFκB, c-Jun, and Hsp27 to the cytokine release nodes, and this PKN was trained to the experimental dataset of both cytokine release and protein phosphorylation. In contrast to the cFL models describing only signaling activation, we found that the family of 141 cFL models fit the cytokine response data with a wider distribution of MSE. The resultant sub-family of seven *filtered* cFL models that fit the data with a MSE less than the average plus one standard deviation of the family MSE is shown. Nodes represent proteins that were either ligand stimulations (green), inhibited (orange), phosphorylation states measured (blue), cytokine secretion measured (yellow) or could not be removed without introducing potential logical inconsistency (white). The grey/black intensity scale of the gates corresponds to the proportion of individual models within the family that include that gate. The graph of the cFL models was generated by a CellNOpt routine using the graphviz visualization engine (www.graphviz.org) followed by manual annotation in Adobe Illustrator.

The resultant models were able to fit the cytokine release data reasonably well (R^2^ = 0.78 for the average predicted by a subset of best-fitting models, Figure S11). Furthermore, the low frequency of several gates in the resultant family of cFL models (Figure S12, Table S3) indicated that, although the promoters of several of the modeled cytokines contained binding sites of transcription factors are known to be modulated by the MEK1/2, GSK3, and CREB pathways (Table S4), activation of these nodes did not predict cytokine release. Thus, we altered our previous PKN by removing the links between these protein signaling and cytokine release nodes and trained it to the data. The resultant family of cFL models (Figure 9) indicated that STAT3 activation explained cytokine release after IL6 stimulation and other signals (Iκb, c-Jun, and Hsp27) explained cytokine release three hours after TNFα or IL1α stimulation.

## Discussion

In this paper, we have described cFL for formal training of a prior knowledge network obtained from a protein signaling network map to experimental data and demonstrated that the ability of cFL to fit intermediate activities was crucial for understanding key features of a biological network. We validated two important biological insights concerning network operation in the HepG2 cells under inflammatory cytokine and growth factor treatment: (i) identification of c-Jun as a downstream locus of crosstalk between growth factor and inflammatory cytokine treatments and (ii) the Ras/Raf/MEK pathway as an avenue for activation of key downstream proteins following exposure of cells to IL6. Both of these insights were dependent on the ability of our cFL models to fit partial protein activation and were thus not appreciated by BL modeling.

We note that the ability of cFL to model intermediate activity data comes at the cost of increased model complexity. This complexity calls into question the identifiability of a cFL model (*i.e.* ability of the CellNOpt-cFL training process to train both parameters and topology given limited data). To address this concern, we considered families of models where each individual model predicted signaling states and the resulting predictions had an average and standard deviation. The standard deviation provided a metric for discrediting predictions for which the models were not constrained. With regard to topology, we considered how often a gate was present in the trained cFL models. This allowed us to determine hypothesized links (those present in the PKN) that were either inconsistent with the data (cFL gates removed from *unprocessed* models) or only marginally important for fitting the data (cFL gates removed from *filtered* models). Thus, the consideration of consensus and variation in an ensemble of models allowed us to account for the non-identifiability of any individual model.

We also illustrated the use of CellNOpt-cFL to (i) predict quantitative phenotypic response data with the same quality as a regression-based approach and (ii) increase the biological understanding of a phenotypic response by generating hypotheses regarding protein signaling pathways that led to cytokine release. Transcriptional and/or non-transcriptional mechanisms could underlie the biological link between the signaling network activation and cytokine release profiles. We investigated predicted and known transcription factor binding sites in the promoters of relevant genes (Table S4), finding that several transcription factors hypothesized by CellNOpt-cFL to drive cytokine release (STAT3 and NFκB) could, in concert with IRF1, potentially lead to the production and secretion of the observed cytokines. Our subsequent test of this notion by qRT-PCR measurement, however, yielded a negative result; expression of the HepG2-secreted proteins were not significantly up-regulated by IL6 stimulation (data not shown). Thus, it appears more likely that non-transcriptional mechanisms, such as exocytosis of secretory vesicles [57], [58] or proteolytic cleavage of pro-forms at the cell plasma membrane [59], [60], was responsible for the cytokine release observations. The persistent development and application of CellNOpt-cFL and complementary methods ([6], [7], [36] and Melas, et al., *submitted*) should continue to deepen our understanding of how signaling networks inform phenotypic responses.

We have shown that CellNOpt-cFL is useful for systematically and quantitatively comparing experimental datasets to a PKN that summarizes decades of dedicated biochemical studies. However, our aim in this work is not to argue for exclusive use of cFL modeling instead of BL or other modeling approaches, but rather to delineate key advantages of cFL modeling for addressing data with intermediate activity values. Training with CellNOpt-cFL is a more difficult optimization problem that is not efficiently solved for networks much larger than those in this work. The BL optimization problem scales as 2*^w^*, where *w* is the number of gates in the processed PKN, whereas the CellNOpt-cFL optimization problem scales as (1+a)*^h^*, where *a* is the number of transfer functions in the set chosen by the genetic algorithm ((1+*a*)≥2; (1+*a*) = 8 as formulated here) and *h* is the number of possible input-output transfer functions in the network (*h*≥*w*). Additionally, as was the case with the reformulation of the BL optimization problem with Integer Linear Programming [30], we acknowledge that there may be more efficient, rigorous ways to solve the optimization problem presented by CellNOpt-cFL.

When training a prior knowledge network to data, we often encountered the need to add links to the prior knowledge network in order to fully describe the data. In this study, this was done manually simply by searching the literature. In the absence of such information, one should automate the process of testing many candidate links. A simple heuristic procedure such as the one we employed for the BL methodology based on mismatches between the best-fit models and data is one option [29]. Alternatively, more complex reverse engineering techniques could be used. The additional complexity of cFL modeling poses significant complications for the implementation of a simple heuristic or reverse engineering technique, but future efforts should investigate best practices for the automation of this process.

An additional prospective application of CellNOpt-cFL is to use a trained cFL model to inform the construction of a model with a different mathematical formalism. One intriguing possibility is that the CellNOpt-cFL methodology might be used to determine topologies to translate into a system of ordinary differential equations (ODEs) with methods such as that presented in [61]. The precise relationship between cFL and ODE parameters is unclear, but the ease of translating from one formalism to the other might be facilitated through the use of continuous AND and OR operators rather than the Min/Max operators utilized in this study. As a first step, we have retrained one of our main results (that presented in Figure 5) using the product of possible outputs to evaluate AND gates and the sum of possible outputs to evaluate OR gates. The models resulting from this procedure (Figure S13) were similar to those obtained previously (Figures 4c, 5), demonstrating the flexibility of this approach to accommodate different AND and OR operators as well as transfer function forms. Such flexibility should aid future attempts to translate CellNOpt-cFL results into other mathematical formalisms.

Finally, the dataset used here was gathered for training a BL model. This dataset was explicitly designed to maximally stimulate or inhibit pathways through the application of saturating doses of ligand and drugs. However, cells *in vivo* face a much more subtle and interesting situation in which ligands are present in combination, often at very different levels. Because cFL can model the graded activation of cell signaling pathways, we suspect that CellNOpt-cFL should prove particularly useful with signaling data collected under more physiological conditions. Our laboratories are currently pursuing experimental studies in this direction.

## Materials and Methods

### Optimization procedure

Model compression and expansion was performed with CellNOpt as previously described [29]. The discrete genetic algorithm in the CellNOpt BL variant was adapted so that discrete variables specified a transfer function rather than the gate type. Because our datasets (toy example and HepG2) only contained saturating concentrations of ligand stimulation, the normalized values of ligand model inputs were one or zero. In this instance, using normalized Hill functions to model interactions downstream of these zero or one inputs would result in all downstream nodes also reaching levels of zero or one (a Boolean simulation). To circumvent this issue, we represented interactions linking a ligand input to a downstream component with linear transfer functions with a y-intercept of zero and possible values of slope of 0.2, 0.3, 0.4, 0.5, 0.6, 0.7, and 0.8 as well as the absence of the interaction. All other interactions were modeled with the normalized Hill function described in Figure 1 where the following transfer functions were possible: gate not active, approximately linear transfer function (n = 1.01, k = 68.5098 chosen for computation efficiency and numerical stability), or sigmoidal transfer function (n = 3) with an EC_50_ of 0.2, 0.3, 0.4, 0.5, 0.6, or 0.7 (Figure S14). These transfer functions were chosen because the models resulting from the training represented many different topologies while still fitting the data well. We found that including a subset of three to five of the aforementioned transfer functions would have also accomplished these goals, but including ten transfer functions resulted in a larger fraction of models that did not fit the data well. This necessitated the addition of a step to choose a subset of well-fitted models from the family of trained models, and this subset did not significantly differ from the family of models obtained with fewer possible transfer functions. Given that more transfer functions allowed us to more accurately represent parameter space, this result implied that the genetic algorithm was converging to poorly-fit local minima because the search space was too large. We therefore concluded that usage of seven transfer functions balanced coverage of search space and ability to identify well-fitting models.

### Sensitivity of a cFL gate

Sensitivity is calculated as (1 – EC_50_) for cFL gates modeled with normalized Hill functions and 0.5*slope for cFL gates modeled with weighted linear transfer functions.

### Calculation of MSE

Mean squared error was calculated with the following formula
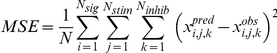
where N is the total number of data points, N_sig_ is the number of protein signals measured, N_stim_ is the number of cytokine or growth factor stimulations, N_inhib_ is the number of inhibition conditions used, and x^pred^_i,j,k_ and x^obs^_i,j,k_ are the predicted and observed level of the i^th^ protein signal under the j^th^ stimulation and k^th^ inhibition condition, respectively. In some cases, only the MSE of a subset of the data points is calculated for more specific error analysis. In these instances, the previous formula holds, but signal and/or stimulation conditions are constant and indicated with subscripts (e.g. MSE_IL6_ is the MSE of all signal measurements under all inhibition conditions and IL6 stimulation).

### Measurement of protein phosphorylation and cytokine release

Protein phosphorylation and cytokine release were measured as described in [36]. Briefly, cells were incubated with small molecule inhibitor before exposure to ligand. Luminex bead-based bioassays were used to determine protein phosphorylation in cell lysate collected immediately before and 30 minutes after ligand exposure. Three hours after ligand exposure, supernatant was collected and Luminex bead-based bioassay used to measure the amount of cytokine that had been secreted.

## Supporting Information

Figure S1 Experimental dataset describing HepG2 signaling response. Each small rectangle represents phosphorylation of the protein indicated on the left at zero and thirty minutes as measured by Luminex bead-based bioassay. HepG2 cells were exposed to the inhibitor indicated below the column and stimulated with the ligand indicated above. Raw intensity (a) and normalized (b) values are shown. Data was normalized as previously described [29] using DataRail software [51]. Briefly, data values below the background or above the saturation signal of the Luminex instrument were not included in the training set (grey fill). The absolute difference between the signal at the time of stimulation and 30 minutes thereafter was divided by the signal at the time stimulation and transformed using a nonlinear Hill transformation. The resulting value was multiplied by a penalty for low values calculated as the Langmuir-transformed ratio of the signal value to its maximum value across all conditions. The resulting value was the normalized value. Plots were generated by the open-source MATLAB toolbox DataRail [51].
(0.28 MB PDF)

Figure S2 Prior knowledge networks (PKNs). PKN0 derived from Ingenuity and used in the BL methodology validation (a, map without purple dashed arrows) was first processed to include two-input AND gates (b) and then used with the CellNOpt-cFL methodology to determine the cFL networks representing this dataset. Results of this analysis led to extension of the PKN to PKN1 (a, purple dashed arrows) which was processed to include either two-input AND gates (PKN1^a^, c) or only include AND gates when an inhibitory interaction was being modeled (PKN1^i^, d). These processed PKNs were then compared to the HepG2 dataset with CellNOpt-cFL. All maps were generated with a CellNOpt routine using the graphviz visualization engine (www.graphviz.org) followed by manual annotation in Adobe Illustrator.
(0.32 MB PDF)

Figure S3 Fit of PKN0 trained to data. PKN0 (Figure S2a) was processed to include all two-input AND gates (Figure S2b) and CellNOpt-cFL used to train 90 network models to the HepG2 dataset (*unprocessed* models are shown). The data is displayed as described in Figure S1, with the exception that the average simulation result is shown with a dashed blue line and the absolute difference in measured and average simulated signal level is indicated with a background color ranging from green (good fit) to red (bad fit). Note that, under the IL1α and IL6 stimulation conditions, many signals are not fit well (as indicated by the red and white coloring). Plots were generated by CellNOpt.
(0.24 MB PDF)

Figure S4 Comparison of MSE and number of parameters of PKN1^a^ and PKN1^i^. The cumulative distribution functions of the MSE and number of final parameters of unprocessed (a,b) and filtered (c,d) models with or without expansion into all plausible two-input AND gates are shown. For both the *unprocessed* and *filtered* models, the error of the models expanded with all plausible two-input AND gates is significantly less than those not fully expanded (p = 4.3x10^-32^ from a Kolmogorov-Smirnov two-sided test of the filtered models). However, both *unprocessed and filtered* models expanded with all plausible two-input gates also contained more parameters than those not fully expanded (p = 3.2×10^−14^ from a Kolmogorov-Smirnov two-sided test of the filtered models). The skewing of the filtered models (d) is due to the heuristic reduction procedure, which sometimes did not remove any parameters from the models.
(0.13 MB PDF)

Figure S5 *Filtered* cFL network models derived from training PKN1 processed to include two-input AND gates (PKN1^a^) to HepG2 dataset. The PKN1 (Figure S2a) was processed to include all two-input AND gates (Figure S2c) and CellNOpt-cFL used to train 191 network models to the HepG2 dataset. Reduction of family of cFL models indicates that cFL AND gates can be removed without greatly affecting the resulting refined model score (a and b; b is a portion of the graph shown in a). The structures of the family of cFL network models trained to the HepG2 dataset are shown (c). Links colored black were present in all models whereas links colored grey were present in a fraction of the models (a darker grey indicates that the cFL gate was present in more models). Filtered cFL network models are shown. Fit to experimental data (d) is displayed as described in Figures S1 and S3. Plots were generated by CellNOpt. Note that, when this PKN1^a^ is used to train the networks, most trained models include the Ras → Map3k1 cFL gate. The inclusion of this link is in contrast to the models obtained when two-input AND gates are only included for inhibitory interactions (Figure 5, Figure S6), where only a few models include this link. This difference is also reflected in the fact that cFL network models processed to include all two-input AND gates are better able to fit data describing c-Jun activation under TGFα stimulation (d compared to Figures S7 and S8). Graphs of cFL network models were generated a CellNOpt routine using the graphviz visualization engine (www.graphviz.org) followed by manual annotation in Adobe Illustrator.
(0.27 MB PDF)

Figure S6 *Unprocessed* cFL network models derived from training PKN1^i^ to HepG2 dataset and investigation of influence of PKN on trained models. a) Structures of the family of unprocessed cFL network models obtained by training the PKN1^i^ (Figure S2d) to the HepG2 dataset. Links colored black were present in all models whereas links colored grey were present in a fraction of the models (a darker grey indicates that the cFL gate was present in more models). These models were compared to the randomization controls, both for the determination of a *p*-value of the models (Table S1) as well as the investigation of the influence of the PKN on the model training process (b,c). The graph of the cFL network models was generated with a CellNOpt routine using the graphviz visualization engine (www.graphviz.org) followed by manual annotation in Adobe Illustrator. (b) We compared *unprocessed* models derived from a PKN with edges randomly added to those derived from the original PKN1^i^. After structure processing (Figure 2 Steps 1–2), a model derived from a PKN with random edges added might have a different number of species as well as interactions than those derived from the original PKN. Thus, to compare these models, we further compressed the networks to include only interactions between the treated, measured, and inhibited species. This treatment allowed us to directly compare models with different intermediate species. When compared to the original PKN1^i^, several edges were added which increased as a function of edges added to the pre-processed PKN, as expected (solid line). For the trained models, we compared edges present frequently in the family of models trained to the original PKN1^i^ (*i.e.* those present in >25% of the models in a.) to those trained to each randomly extended PKN (dashed line). The fraction of different edges in the structures of the trained randomly extended models to those trained to the original PKN1^i^ increased slightly with increasing number of edges added randomly. (c) Comparing between the randomly extended PKNs and models derived from them, connections between treated, measured, and inhibited species that were in the randomly extended PKN but not the original PKN were often but not always removed during the training process. This is to be expected, as not all of the randomly added edges would not be inconsistent with the data, and some might allow the models to fit the data better than the original PKN.
(0.21 MB PDF)

Figure S7 Fit of cFL networks trained using the extended PKN1^i^. The extended prior knowledge network (Figure S2a) was processed to include all two-input AND gates only when an inhibitory interaction was modeled (Figure S2d) and CellNOpt-cFL used to train 243 network models to the HepG2 dataset. The data is displayed as described in Figures S1 and S3. Plots were generated by CellNOpt.
(0.23 MB PDF)

Figure S8 Analysis of systematic error in c-Jun under TGFα stimulation. Both the training and follow-up datasets indicate that c-Jun but not JNK is phosphorylated upon TGFα stimulation. In PKN1, the only path for c-Jun activation is by JNK activation. The cFL networks account for this discrepancy in one of two ways: (1) Partial activation of the JNK node (increasing error) and amplification of this signal to further activate the c-Jun node (decreasing error). CFL networks that followed this treatment contained Ras → MAP3K1 or PI3K → MAP3K1 links (blue “With Crosstalk” case). (2) No activation of c-Jun under TGFα stimulation, increasing error in only the c-Jun signaling node. CFL networks that followed this treatment contained neither Ras → MAP3K1 nor PI3K → MAP3K1 links (red “Without Crosstalk” case). No significant differences in ability to fit the other signals are observed. Each of these treatments of c-Jun activation corresponds to a different biological explanation. The first treatment corresponds to the explanation validated by further experiments (Figure 6a) that JNK was partially activated but our measurement did not reflect this while the second treatment corresponds to the explanation that an interaction we did not include in PKN1 was causing c-Jun to be activated.
(0.08 MB PDF)

Figure S9 Fit of cFL networks to follow up data. (a) Experimental design of follow-up dataset describing the HepG2 response to combinations of ligand and inhibition treatments. (b) Raw data was rescaled using common conditions as described in Prill et al., *in preparation*. (see http://wiki.c2b2.columbia.edu/dream/data/scripts/DREAM4/ for Challenge_3 data scaling scripts). Briefly, a linear correlation the log-normalized signals under common conditions of the training and validation data was fit. Parameters of this line were used to scale the log-normalized validation data, which was then transformed back into the linear range. The resulting rescaled values are shown. (c) CFL networks were trained to the HepG2 dataset using PKN1^i^. The data is displayed as described in Figures S1 and S3. The filtered models were able to fit the validation data with an MSE of 0.076±0.005. Some of this error (∼13%) was expected, as these conditions were similar to the experimental conditions under which the main discrepancies between the training data and models were observed (phosphorylation of IRS1s and p70s6 under IL1α stimulation and MEK inhibition). An additional ∼25% of the error can be accounted for by variation in the normalized data of the common conditions of the two datasets. Plots were generated by CellNOpt (fit to data) and the open-source MATLAB toolbox DataRail [51] (raw data).
(0.36 MB PDF)

Figure S10 Fit of PLSR model of phenotypic cytokine release data. A three-component PLSR model fit normalized cytokine release data well in most cases except the condition of TNFα stimulation and Iκb inhibition. The data is displayed as described in and S3. Plots were generated by CellNOpt.
(0.13 MB PDF)

Figure S11 Fit of cFL models linking protein signals to phenotypic cytokine release. Several signaling nodes (MEK1/2, CREB, GSK3, c-Jun, Hsp27, I κb, and STAT3) were linked to cytokine release nodes (IL1β, IL4, GCSF, IFN γ, and SDF1 α) in an extended PKN (Table 3, PKN2D) and trained to the HepG2 dataset of both protein signaling and cytokine release data. The fit of the family of cFL models (a) was similar to other cFL models for the signaling data but slightly worse than the PLSR model fit to cytokine release data (Figure S10). A subset of these models had MSEs less than one standard deviation of the mean MSE of the family of models. Those models were deemed most reliable because they fit the data very well. The fit of the average prediction of these models is shown in (b). These average structure for this subset can be found in Figure S12. The data is displayed as described in Figures S1 and S3. Plots were generated by CellNOpt.
(0.41 MB PDF)

Figure S12 Structure of filtered cFL models linking protein signals to phenotypic cytokine release. Several signaling nodes (MEK1/2, CREB, GSK3, c-Jun, Hsp27, Iκb, and STAT3) were linked to cytokine release nodes (IL1β, IL4, G-CSF, IFNγ and SDF1α) in an extended prior knowledge network (Table 3, PKN2D) and trained to the HepG2 dataset of both protein signaling and cytokine release data. Structures of the subset of 31 filtered cFL network models with MSE less than one standard deviation from the mean of the entire family is shown. Links colored black were present in all models whereas links colored grey were present in a fraction of the models (a darker grey indicates that the cFL gate was present in more models). Graph of cFL network models was generated by a CellNOpt routine using the graphviz visualization engine (www.graphviz.org) followed by manual annotation in Adobe Illustrator. Because few cFL network models contained links between MEK1/2, CREB, and GSK3 to cytokine release (Table S3), these links were removed from the extended prior knowledge network and the resultant network trained to the data. The average prediction of these models fit similarly to those in Figure S11 and the models' structures can be found in Figure 9.
(0.14 MB PDF)

Figure S13 Investigating the use of alternate mathematical operators to evaluate AND and OR gates. The extended prior knowledge network (Figure S2a) was processed to include all two-input AND gates only when an inhibitory interaction was modeled (Figure S2d, PKN1^i^) and CellNOpt-cFL used to train 149 network models to the HepG2 dataset. However, the cFL formalism was altered slightly so that an AND gate was evaluated using the product operator and an OR operation evaluated with the sum operator, where the scaling was maintained to between zero and one by limiting the maximum value of any species to one. Note the similarity of these results to those obtained with Min/Max operators are used to evaluate AND and OR gates, respectively (compare Figure 4c to part a of this figure, Figure 5 to part b, and Figure 7 to part c). Reduction of the family of cFL models indicates that a selection threshold of 0.005 is also appropriate in this case. The structures of the family of cFL network models trained to the HepG2 dataset are shown (b). Links colored black were present in all models whereas links colored grey were present in a fraction of the models (a darker grey indicates that the cFL gate was present in more models). Filtered cFL network models are shown. Fit to experimental data (c) is displayed as described in Figures S1 and S3. Plots were generated by CellNOpt.
(0.24 MB PDF)

Figure S14 Transfer functions included in the discrete genetic algorithm optimization process. The discrete genetic algorithm chose one of the transfer functions with the indicated parameter sets during the optimization process to relate each input species' value to the output species' value. (a) Transfer functions used to relate species within the network. (b) Transfer functions used to relate ligand input values to the species immediately downstream of them.
(0.14 MB PDF)

Table S1 Assessing statistical significance of cFL models derived from PKN1^i^. network randomization were performed. In “Swap Heads” randomization, the input of each interaction was randomly exchanged with the input of another interaction while in “Swap Tails,” this process was executed for outputs of each interaction. “Swap Inputs” randomization involved swapping the inputs of all interactions with a randomly chosen output node with the inputs of all interactions with another randomly chosen output node. Finally, completely random networks were generated with the same number of nodes and edges as the extended prior-knowledge network, at least one edge per node, and no incoming but at least one outgoing edge for each network input [29]. For the random data case, P-Values were calculated for each model trained to the real dataset using the Z-score of the model MSE compared to the distribution of randomized data models' MSEs. For the random networks case, the distribution of MSEs was not normal as assessed by the Jarque-Bera test at α≥0.001. In this case, P-value was calculated as the instance of random models with score less than that of the trained model, of which no instance was observed for any model.
(0.07 MB PDF)

Table S2 Test sets for cross validation experiment. In each test case, the measured signal under one stimulation condition with all inhibitor conditions was used as the test data. The remaining data was training data.
(0.08 MB PDF)

Table S3 Frequency of interactions linking protein signals to phenotypic cytokine release. Frequency of links in the subset of 31 cFL models (Figure S12) with MSEs lower than one standard deviation of the family of models.
(0.08 MB PDF)

Table S4 Experimentally verified and computationally predicted transcription factor binding sites in relevant genes. Genes were queried in BioBase TRANSFAC [62], [63] for experimentally verified or computationally predicted transcription factor binding sites and the March 2006 (NCBI36/hg18) assembly of UCSC Genome Bioinformatics (http://genome.ucsc.edu/) for computationally predicted transcription factor binding sites. Those binding sites listed below were included either because they were binding sites of phosphorylated proteins measured or transcription factors modulated by phosphorylated proteins measured.
(0.09 MB PDF)

Text S1 Supplementary materials and methods.
(0.09 MB PDF)
