# Supplementary figures and images for: Training Signaling Pathway Maps to Biochemical Data with Constrained Fuzzy Logic: Quantitative Analysis of Liver Cell Responses to Inflammatory Stimuli
Source: PLoS Comput Biol. 2011 Mar 3;7(3):e1001099. doi: 10.1371/journal.pcbi.1001099 (PMC3048376; doi:10.1371/journal.pcbi.1001099)

a)

## Ligand Stimulation

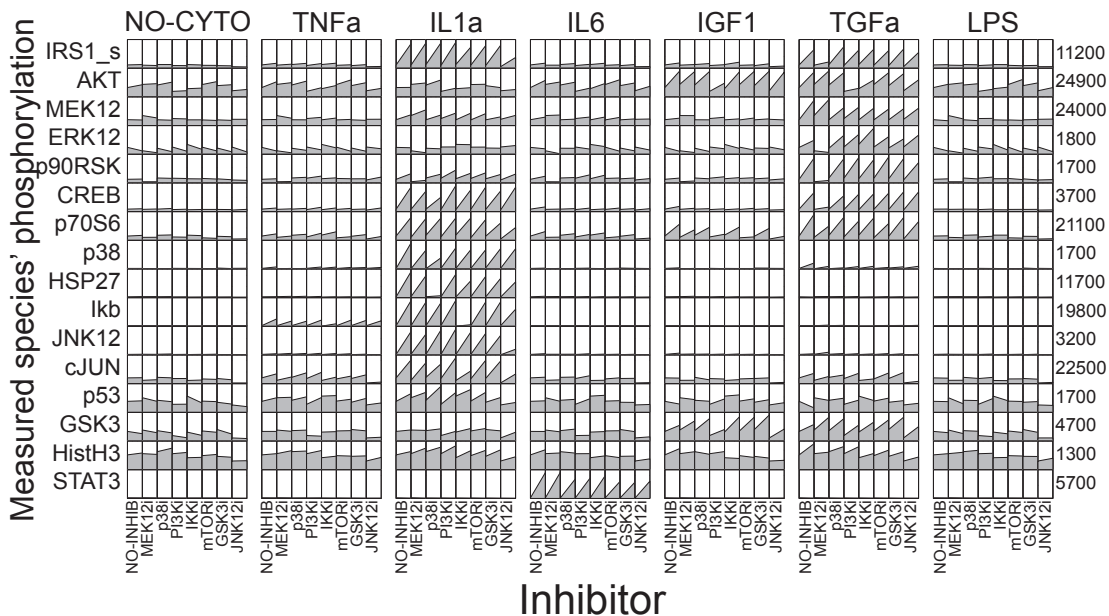

b)

## Ligand Stimulation

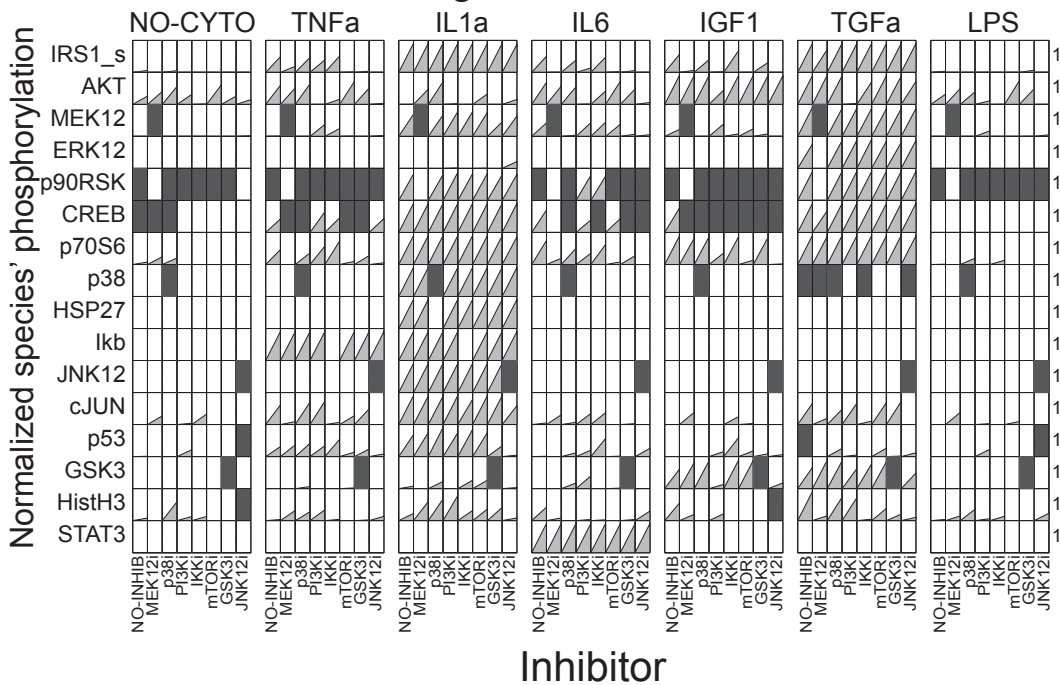

Supplement: Figure S1 — Experimental dataset describing HepG2 signaling response. Each small rectangle represents phosphorylation of the protein indicated on the left at zero and thirty minutes as measured by Luminex bead-based bioassay. HepG2 cells were exposed to the inhibitor indicated below the column and stimulated with the ligand indicated above. Raw intensity (a) and normalized (b) values are shown. Data was normalized as previously described [29] using DataRail software [51]. Briefly, data values below the background or above the saturation signal of the Luminex instrument were not included in the training set (grey fill). The absolute difference between the signal at the time of stimulation and 30 minutes thereafter was divided by the signal at the time stimulation and transformed using a nonlinear Hill transformation. The resulting value was multiplied by a penalty for low values calculated as the Langmuir-transformed ratio of the signal value to its maximum value across all conditions. The resulting value was the normalized value. Plots were generated by the open-source MATLAB toolbox DataRail [51]. (0.28 MB PDF) [file pcbi.1001099.s001.pdf]

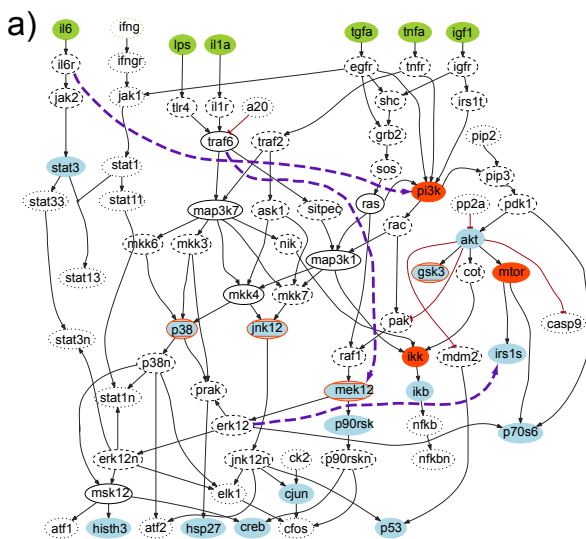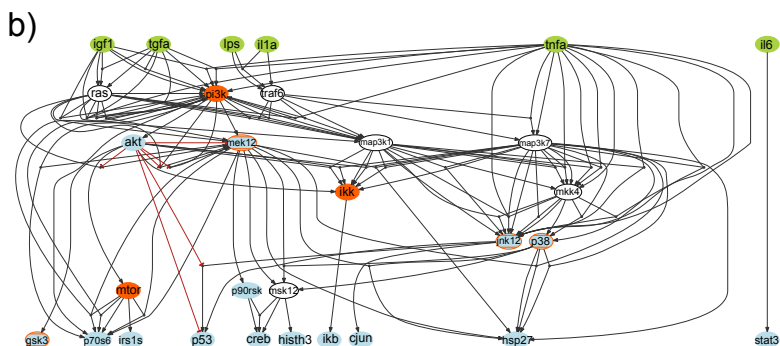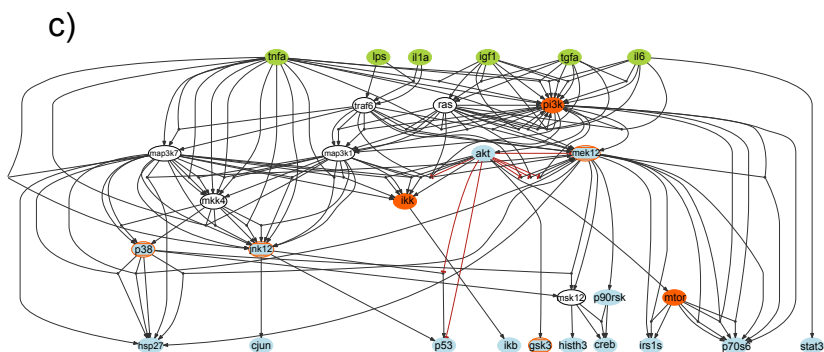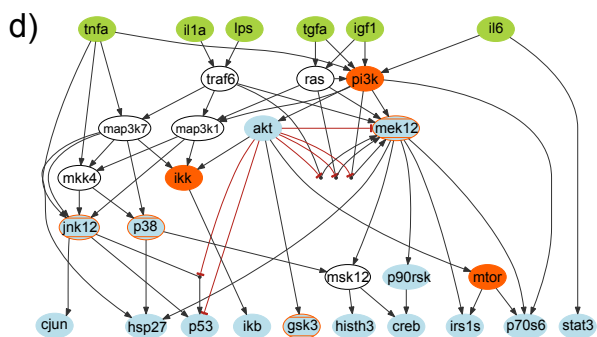

Supplement: Figure S2 — Prior knowledge networks (PKNs). PKN0 derived from Ingenuity and used in the BL methodology validation (a, map without purple dashed arrows) was first processed to include two-input AND gates (b) and then used with the CellNOpt-cFL methodology to determine the cFL networks representing this dataset. Results of this analysis led to extension of the PKN to PKN1 (a, purple dashed arrows) which was processed to include either two-input AND gates (PKN1a, c) or only include AND gates when an inhibitory interaction was being modeled (PKN1i, d). These processed PKNs were then compared to the HepG2 dataset with CellNOpt-cFL. All maps were generated with a CellNOpt routine using the graphviz visualization engine (www.graphviz.org) followed by manual annotation in Adobe Illustrator. (0.32 MB PDF) [file pcbi.1001099.s002.pdf]

# Ligand Stimulation

Species' phosphorylation

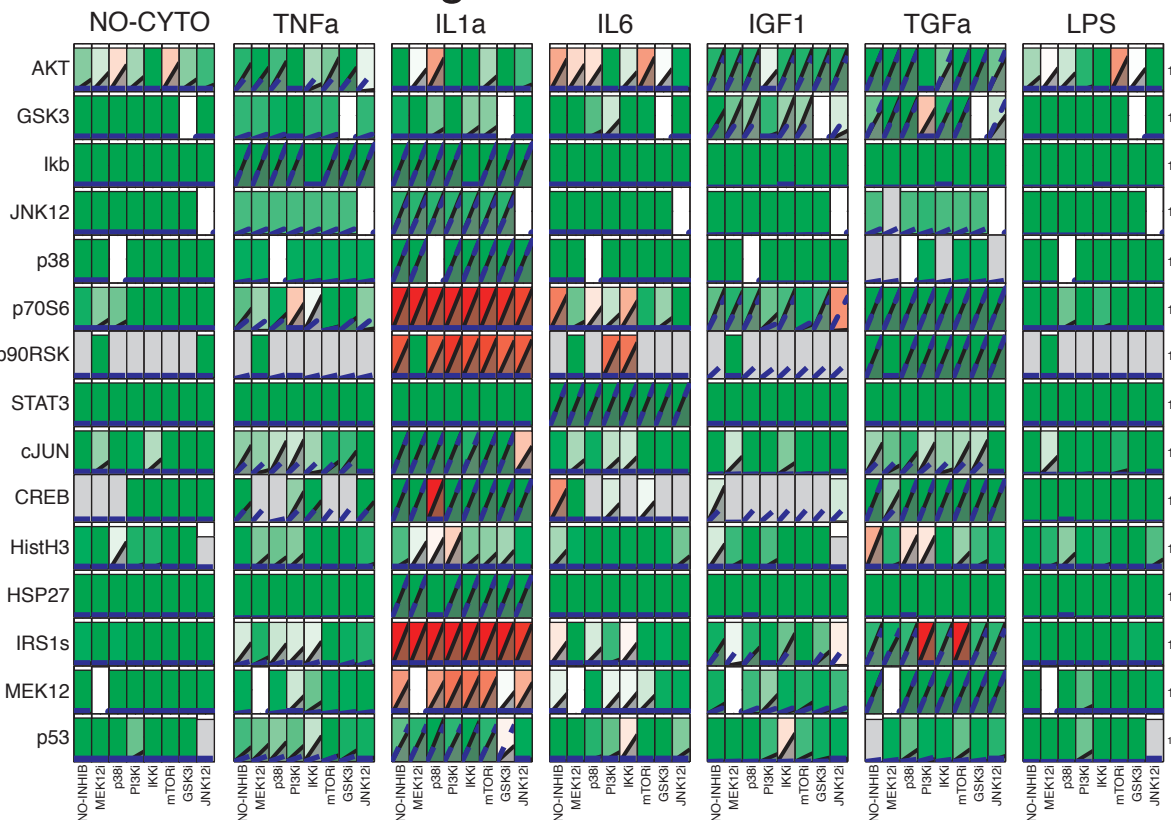

Inhibitor

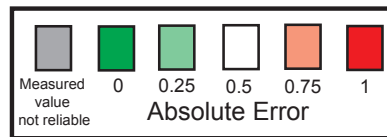

Supplement: Figure S3 — Fit of PKN0 trained to data. PKN0 (Figure S2a) was processed to include all two-input AND gates (Figure S2b) and CellNOpt-cFL used to train 90 network models to the HepG2 dataset (unprocessed models are shown). The data is displayed as described in Figure S1, with the exception that the average simulation result is shown with a dashed blue line and the absolute difference in measured and average simulated signal level is indicated with a background color ranging from green (good fit) to red (bad fit). Note that, under the IL1α and IL6 stimulation conditions, many signals are not fit well (as indicated by the red and white coloring). Plots were generated by CellNOpt. (0.24 MB PDF) [file pcbi.1001099.s003.pdf]

MSEs

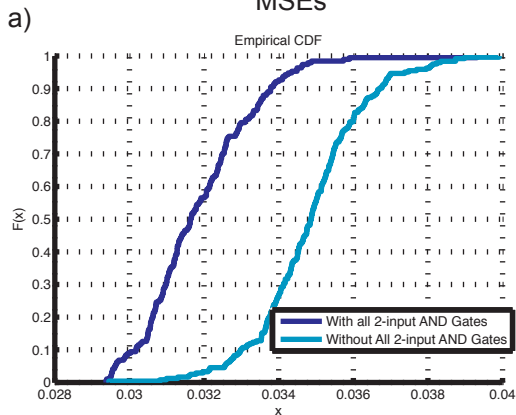

Number of Parameters

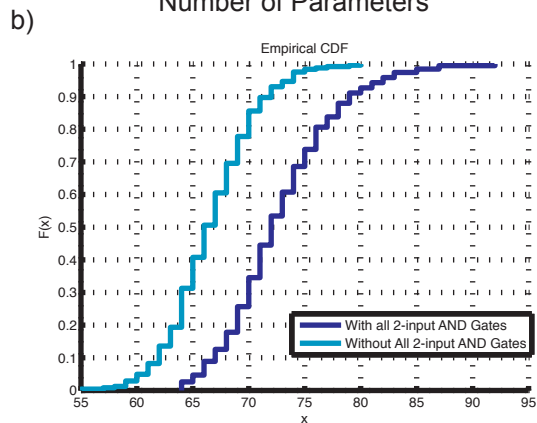

Filtered Models

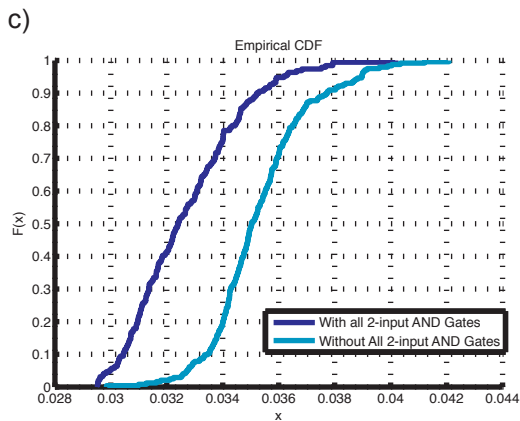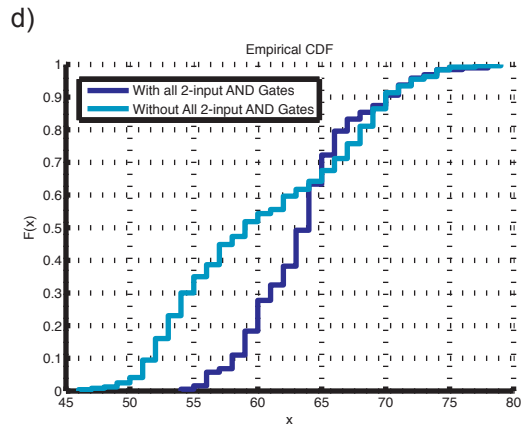

Supplement: Figure S4 — Comparison of MSE and number of parameters of PKN1a and PKN1i. The cumulative distribution functions of the MSE and number of final parameters of unprocessed (a,b) and filtered (c,d) models with or without expansion into all plausible two-input AND gates are shown. For both the unprocessed and filtered models, the error of the models expanded with all plausible two-input AND gates is significantly less than those not fully expanded (p = 4.3x10^-32^ from a Kolmogorov-Smirnov two-sided test of the filtered models). However, both unprocessed and filtered models expanded with all plausible two-input gates also contained more parameters than those not fully expanded (p = 3.2×10^−14^ from a Kolmogorov-Smirnov two-sided test of the filtered models). The skewing of the filtered models (d) is due to the heuristic reduction procedure, which sometimes did not remove any parameters from the models. (0.13 MB PDF) [file pcbi.1001099.s004.pdf]

# Species' phosphorylation

## Ligand Stimulation

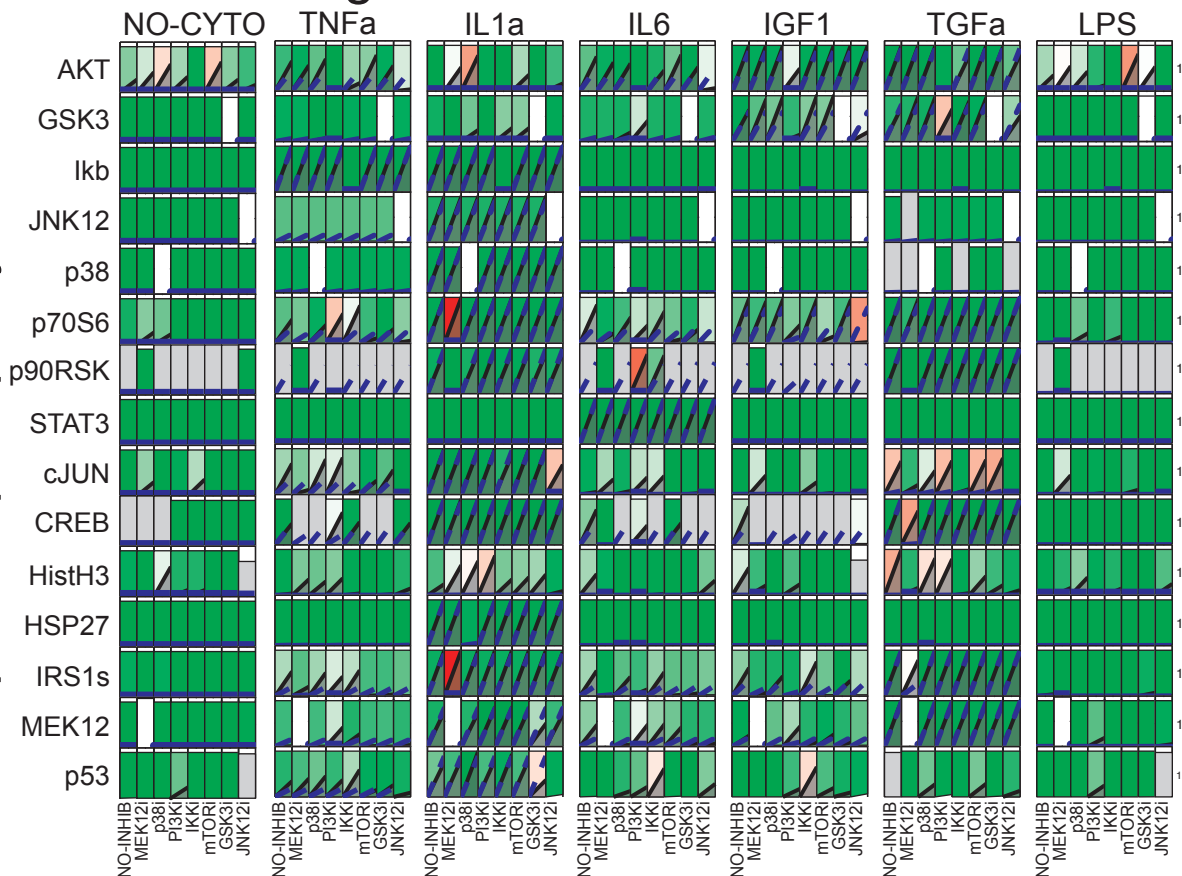

## Inhibitor

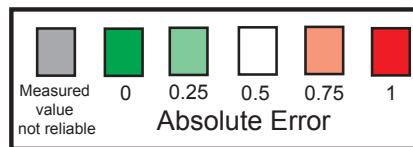

Supplement: Figure S7 — Fit of cFL networks trained using the extended PKN1i. The extended prior knowledge network (Figure S2a) was processed to include all two-input AND gates only when an inhibitory interaction was modeled (Figure S2d) and CellNOpt-cFL used to train 243 network models to the HepG2 dataset. The data is displayed as described in Figures S1 and S3. Plots were generated by CellNOpt. (0.23 MB PDF) [file pcbi.1001099.s007.pdf]

Average MSE

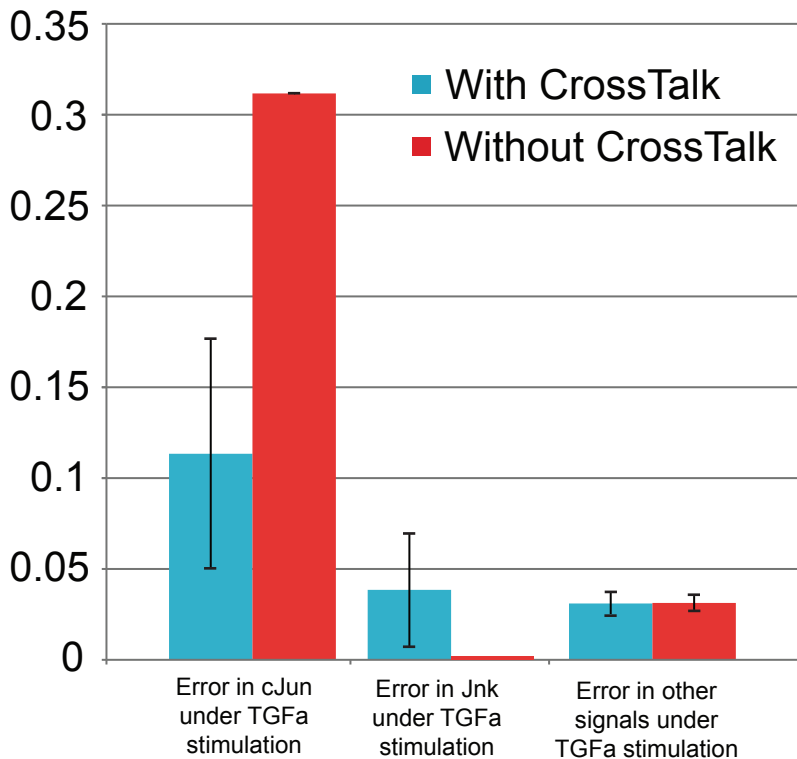

Supplement: Figure S8 — Analysis of systematic error in c-Jun under TGFα stimulation. Both the training and follow-up datasets indicate that c-Jun but not JNK is phosphorylated upon TGFα stimulation. In PKN1, the only path for c-Jun activation is by JNK activation. The cFL networks account for this discrepancy in one of two ways: (1) Partial activation of the JNK node (increasing error) and amplification of this signal to further activate the c-Jun node (decreasing error). CFL networks that followed this treatment contained Ras → MAP3K1 or PI3K → MAP3K1 links (blue “With Crosstalk” case). (2) No activation of c-Jun under TGFα stimulation, increasing error in only the c-Jun signaling node. CFL networks that followed this treatment contained neither Ras → MAP3K1 nor PI3K → MAP3K1 links (red “Without Crosstalk” case). No significant differences in ability to fit the other signals are observed. Each of these treatments of c-Jun activation corresponds to a different biological explanation. The first treatment corresponds to the explanation validated by further experiments (Figure 6a) that JNK was partially activated but our measurement did not reflect this while the second treatment corresponds to the explanation that an interaction we did not include in PKN1 was causing c-Jun to be activated. (0.08 MB PDF) [file pcbi.1001099.s008.pdf]

a)

Experimental Design

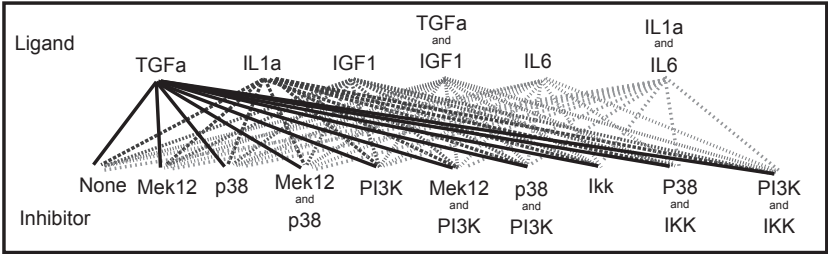

b)

Ligand Stimulation

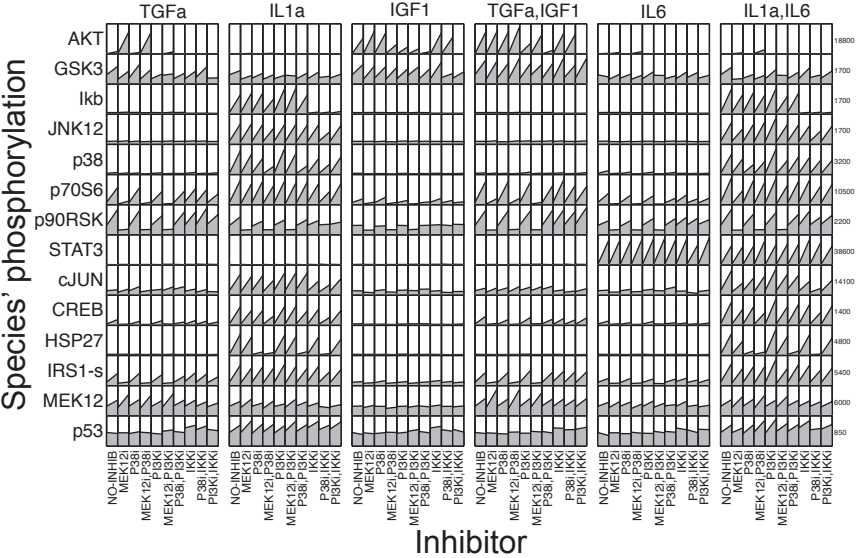

c)

Ligand Stimulation

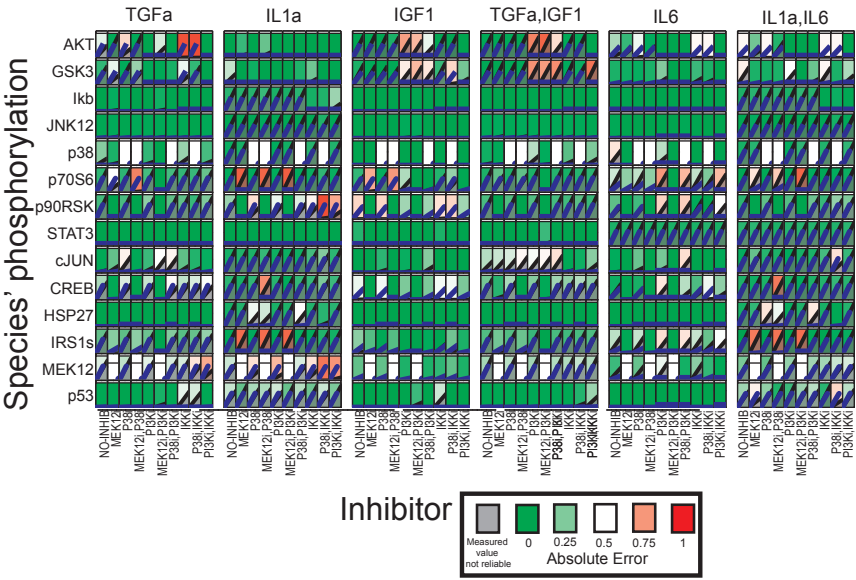

Supplement: Figure S9 — Fit of cFL networks to follow up data. (a) Experimental design of follow-up dataset describing the HepG2 response to combinations of ligand and inhibition treatments. (b) Raw data was rescaled using common conditions as described in Prill et al., in preparation. (see http://wiki.c2b2.columbia.edu/dream/data/scripts/DREAM4/ for Challenge_3 data scaling scripts). Briefly, a linear correlation the log-normalized signals under common conditions of the training and validation data was fit. Parameters of this line were used to scale the log-normalized validation data, which was then transformed back into the linear range. The resulting rescaled values are shown. (c) CFL networks were trained to the HepG2 dataset using PKN1i. The data is displayed as described in Figures S1 and S3. The filtered models were able to fit the validation data with an MSE of 0.076±0.005. Some of this error (∼13%) was expected, as these conditions were similar to the experimental conditions under which the main discrepancies between the training data and models were observed (phosphorylation of IRS1s and p70s6 under IL1α stimulation and MEK inhibition). An additional ∼25% of the error can be accounted for by variation in the normalized data of the common conditions of the two datasets. Plots were generated by CellNOpt (fit to data) and the open-source MATLAB toolbox DataRail [51] (raw data). (0.36 MB PDF) [file pcbi.1001099.s009.pdf]

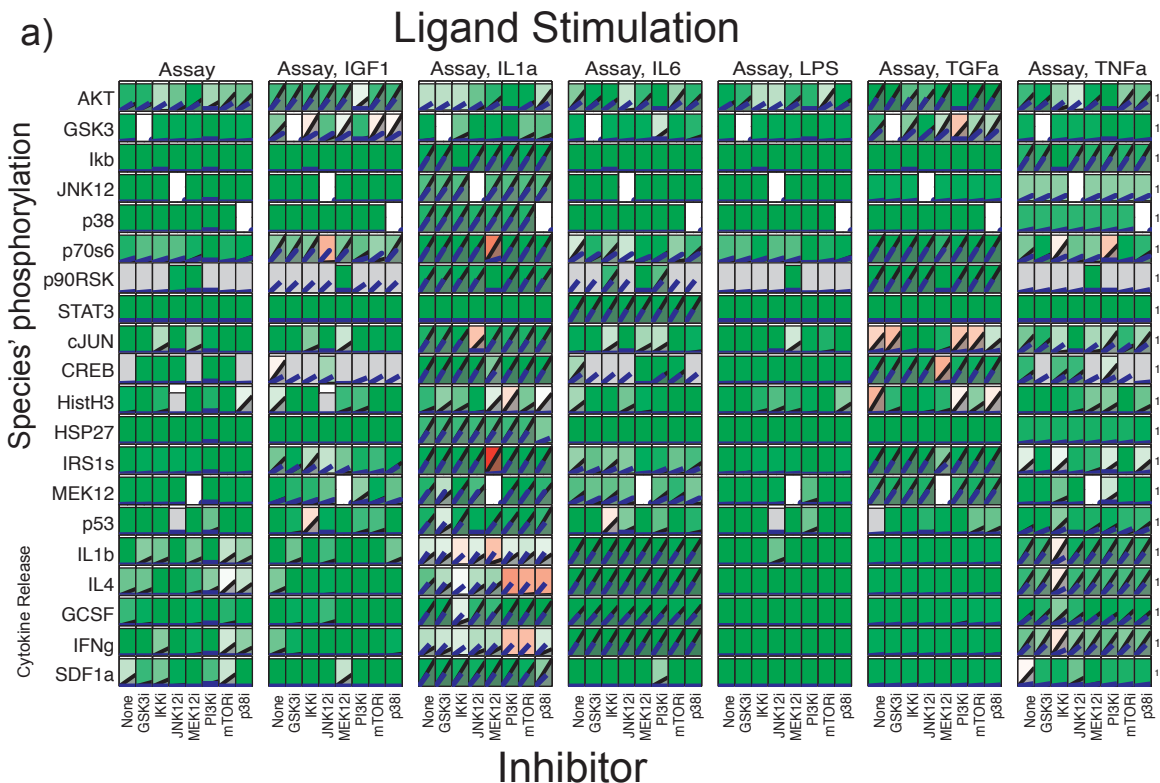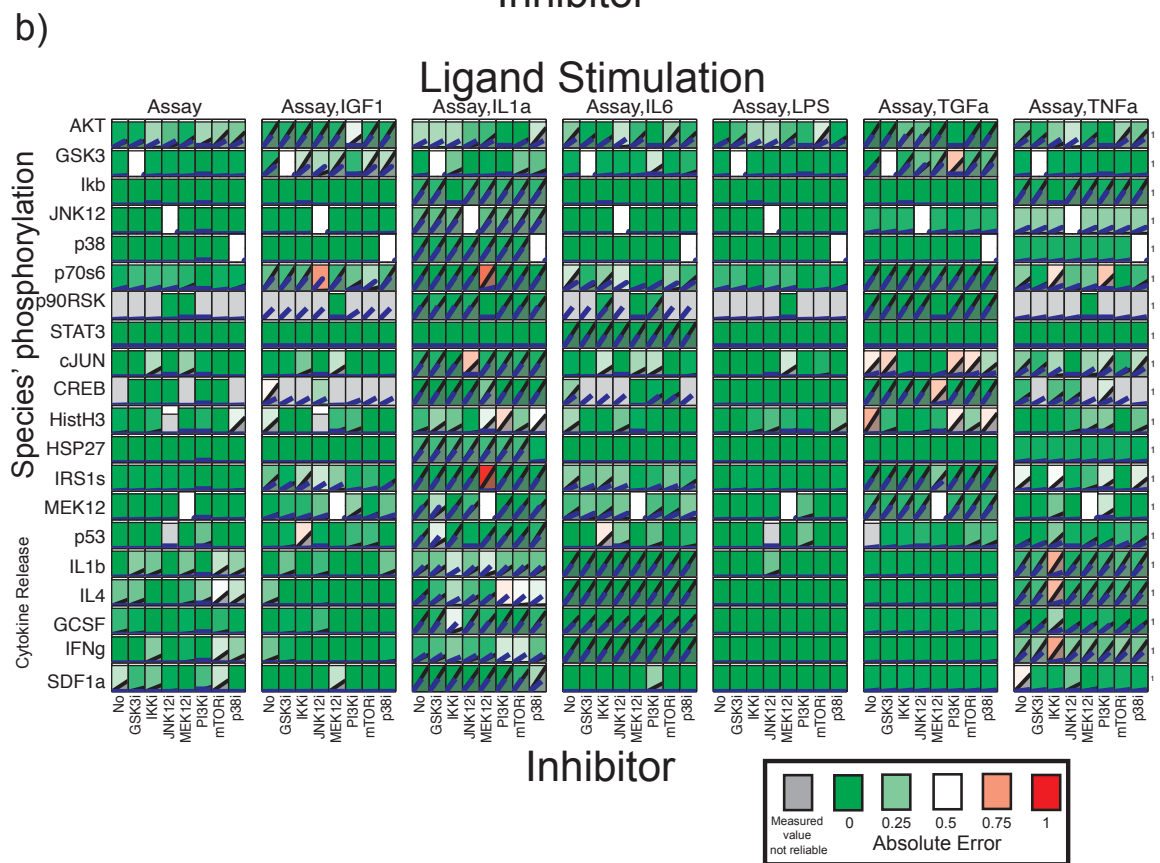

Supplement: Figure S11 — Fit of cFL models linking protein signals to phenotypic cytokine release. Several signaling nodes (MEK1/2, CREB, GSK3, c-Jun, Hsp27, I κb, and STAT3) were linked to cytokine release nodes (IL1β, IL4, GCSF, IFN γ, and SDF1 α) in an extended PKN (Table 3, PKN2D) and trained to the HepG2 dataset of both protein signaling and cytokine release data. The fit of the family of cFL models (a) was similar to other cFL models for the signaling data but slightly worse than the PLSR model fit to cytokine release data (Figure S10). A subset of these models had MSEs less than one standard deviation of the mean MSE of the family of models. Those models were deemed most reliable because they fit the data very well. The fit of the average prediction of these models is shown in (b). These average structure for this subset can be found in Figure S12. The data is displayed as described in Figures S1 and S3. Plots were generated by CellNOpt. (0.41 MB PDF) [file pcbi.1001099.s011.pdf]

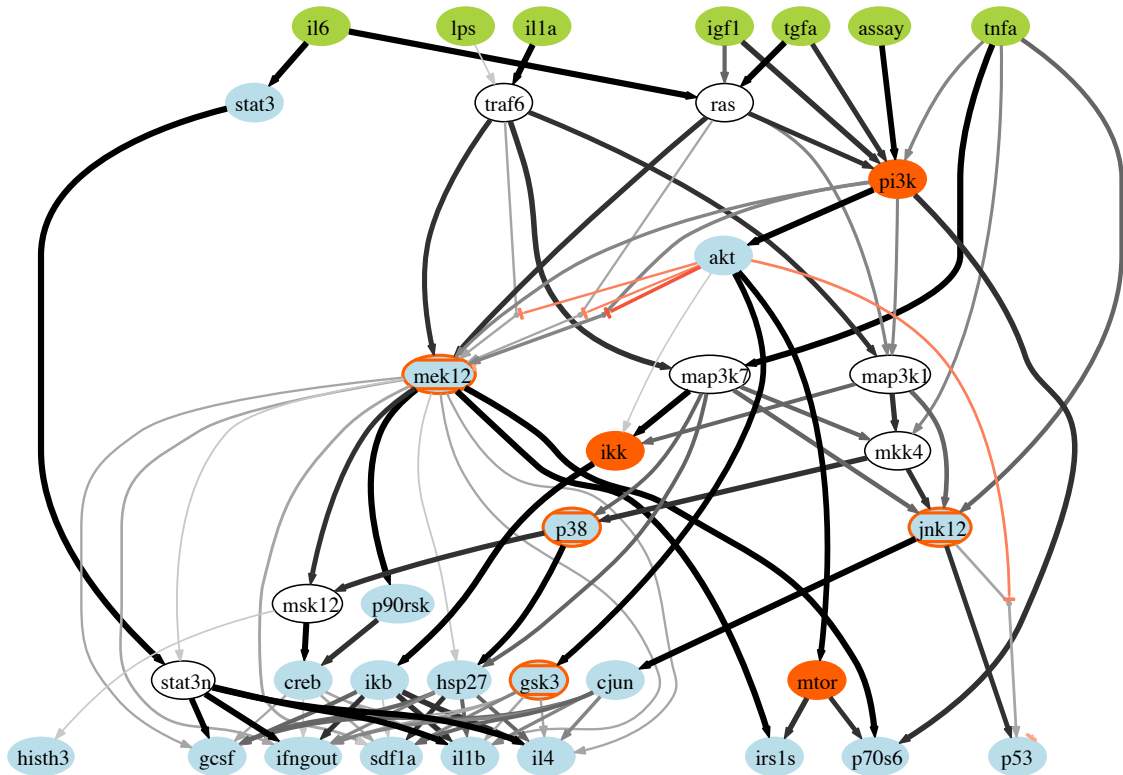

Supplement: Figure S12 — Structure of filtered cFL models linking protein signals to phenotypic cytokine release. Several signaling nodes (MEK1/2, CREB, GSK3, c-Jun, Hsp27, Iκb, and STAT3) were linked to cytokine release nodes (IL1β, IL4, G-CSF, IFNγ and SDF1α) in an extended prior knowledge network (Table 3, PKN2D) and trained to the HepG2 dataset of both protein signaling and cytokine release data. Structures of the subset of 31 filtered cFL network models with MSE less than one standard deviation from the mean of the entire family is shown. Links colored black were present in all models whereas links colored grey were present in a fraction of the models (a darker grey indicates that the cFL gate was present in more models). Graph of cFL network models was generated by a CellNOpt routine using the graphviz visualization engine (www.graphviz.org) followed by manual annotation in Adobe Illustrator. Because few cFL network models contained links between MEK1/2, CREB, and GSK3 to cytokine release (Table S3), these links were removed from the extended prior knowledge network and the resultant network trained to the data. The average prediction of these models fit similarly to those in Figure S11 and the models' structures can be found in Figure 9. (0.14 MB PDF) [file pcbi.1001099.s012.pdf]

a)

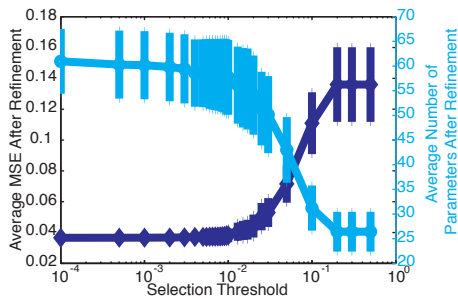

b)

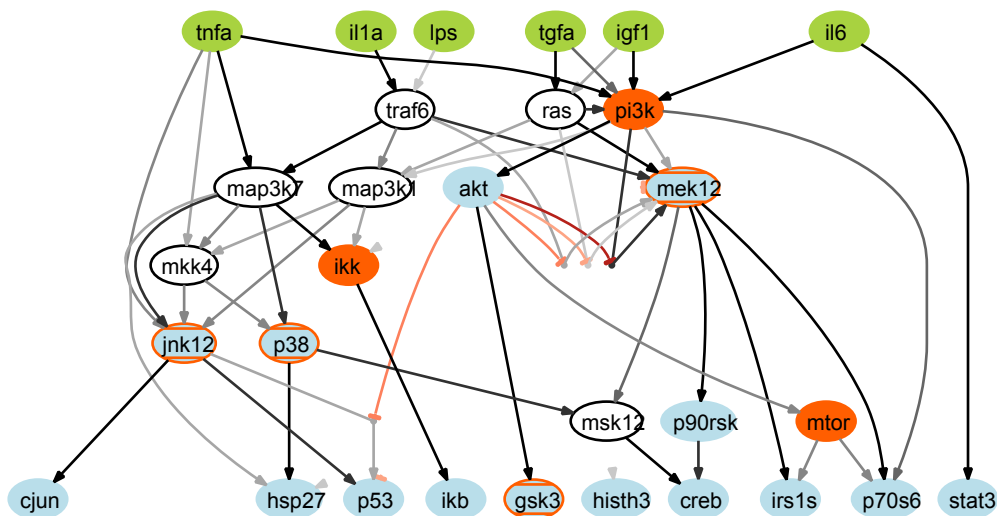

c)

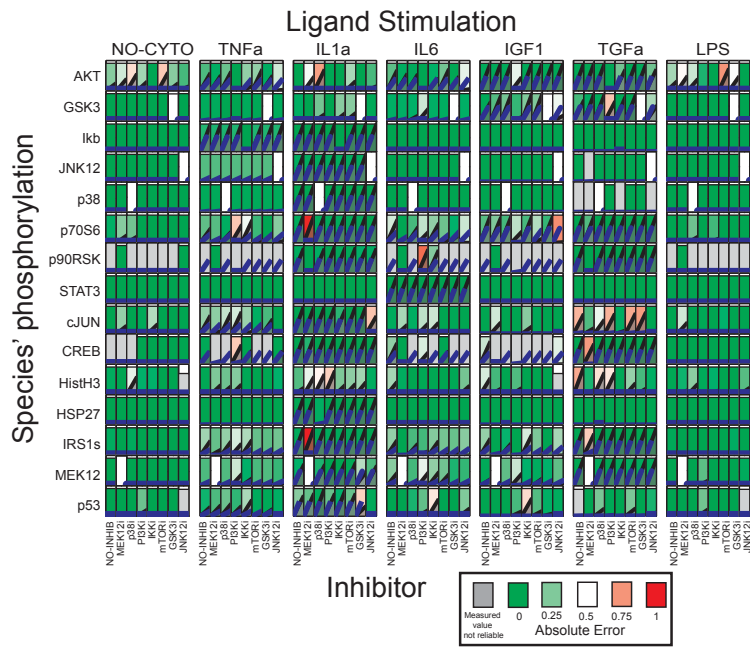

Supplement: Figure S13 — Investigating the use of alternate mathematical operators to evaluate AND and OR gates. The extended prior knowledge network (Figure S2a) was processed to include all two-input AND gates only when an inhibitory interaction was modeled (Figure S2d, PKN1i) and CellNOpt-cFL used to train 149 network models to the HepG2 dataset. However, the cFL formalism was altered slightly so that an AND gate was evaluated using the product operator and an OR operation evaluated with the sum operator, where the scaling was maintained to between zero and one by limiting the maximum value of any species to one. Note the similarity of these results to those obtained with Min/Max operators are used to evaluate AND and OR gates, respectively (compare Figure 4c to part a of this figure, Figure 5 to part b, and Figure 7 to part c). Reduction of the family of cFL models indicates that a selection threshold of 0.005 is also appropriate in this case. The structures of the family of cFL network models trained to the HepG2 dataset are shown (b). Links colored black were present in all models whereas links colored grey were present in a fraction of the models (a darker grey indicates that the cFL gate was present in more models). Filtered cFL network models are shown. Fit to experimental data (c) is displayed as described in Figures S1 and S3. Plots were generated by CellNOpt. (0.24 MB PDF) [file pcbi.1001099.s013.pdf]

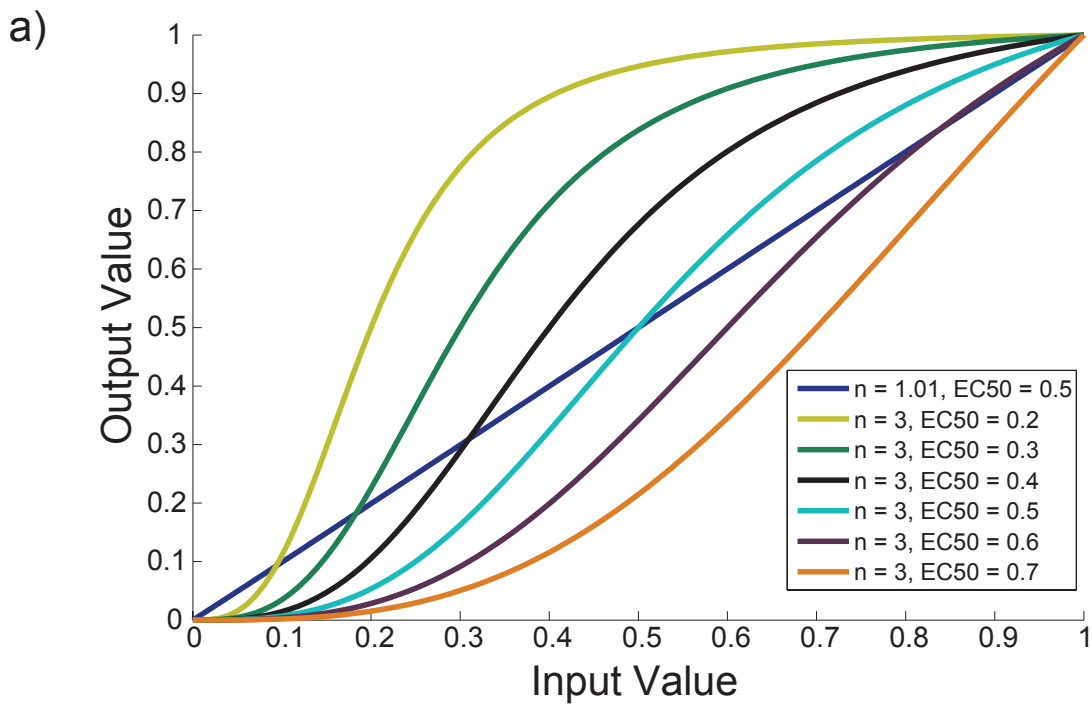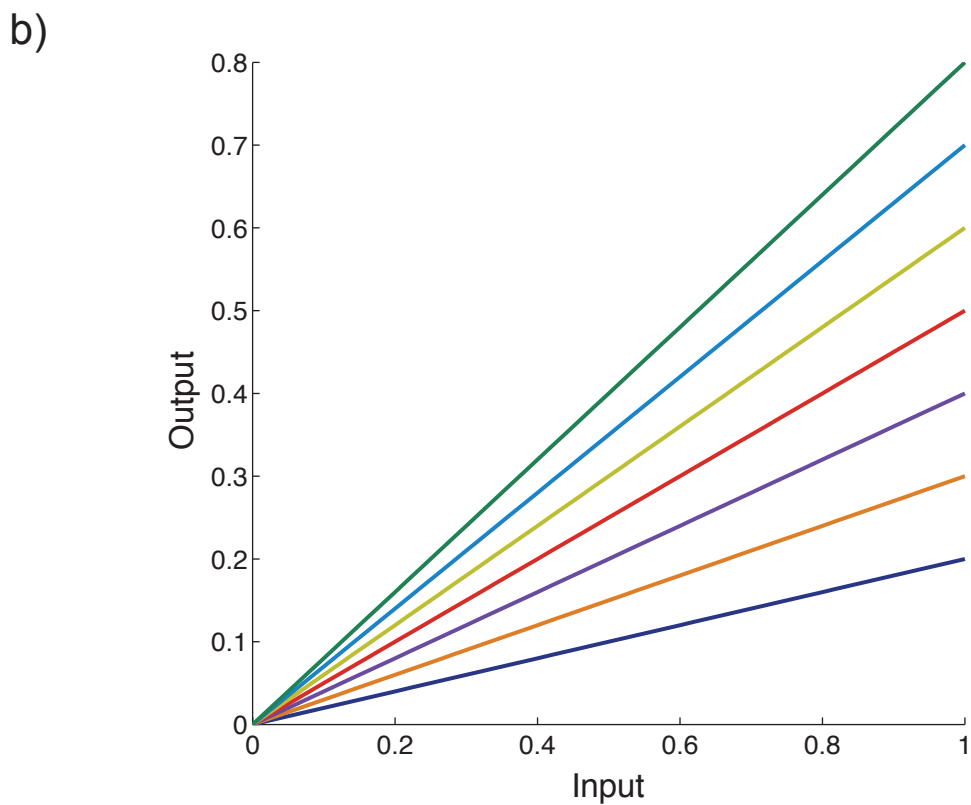

Supplement: Figure S14 — Transfer functions included in the discrete genetic algorithm optimization process. The discrete genetic algorithm chose one of the transfer functions with the indicated parameter sets during the optimization process to relate each input species' value to the output species' value. (a) Transfer functions used to relate species within the network. (b) Transfer functions used to relate ligand input values to the species immediately downstream of them. (0.14 MB PDF) [file pcbi.1001099.s014.pdf]
