# Supplementary material for: Training Signaling Pathway Maps to Biochemical Data with Constrained Fuzzy Logic: Quantitative Analysis of Liver Cell Responses to Inflammatory Stimuli
Source: PLoS Comput Biol. 2011 Mar 3;7(3):e1001099. doi: 10.1371/journal.pcbi.1001099 (PMC3048376; doi:10.1371/journal.pcbi.1001099)
Supplement: Figure S6 — Unprocessed cFL network models derived from training PKN1i to HepG2 dataset and investigation of influence of PKN on trained models. a) Structures of the family of unprocessed cFL network models obtained by training the PKN1i (Figure S2d) to the HepG2 dataset. Links colored black were present in all models whereas links colored grey were present in a fraction of the models (a darker grey indicates that the cFL gate was present in more models). These models were compared to the randomization controls, both for the determination of a p-value of the models (Table S1) as well as the investigation of the influence of the PKN on the model training process (b,c). The graph of the cFL network models was generated with a CellNOpt routine using the graphviz visualization engine (www.graphviz.org) followed by manual annotation in Adobe Illustrator. (b) We compared unprocessed models derived from a PKN with edges randomly added to those derived from the original PKN1i. After structure processing (Figure 2 Steps 1–2), a model derived from a PKN with random edges added might have a different number of species as well as interactions than those derived from the original PKN. Thus, to compare these models, we further compressed the networks to include only interactions between the treated, measured, and inhibited species. This treatment allowed us to directly compare models with different intermediate species. When compared to the original PKN1i, several edges were added which increased as a function of edges added to the pre-processed PKN, as expected (solid line). For the trained models, we compared edges present frequently in the family of models trained to the original PKN1i (i.e. those present in >25% of the models in a.) to those trained to each randomly extended PKN (dashed line). The fraction of different edges in the structures of the trained randomly extended models to those trained to the original PKN1i increased slightly with increasing number of edges added randomly. ( [file pcbi.1001099.s006.pdf]

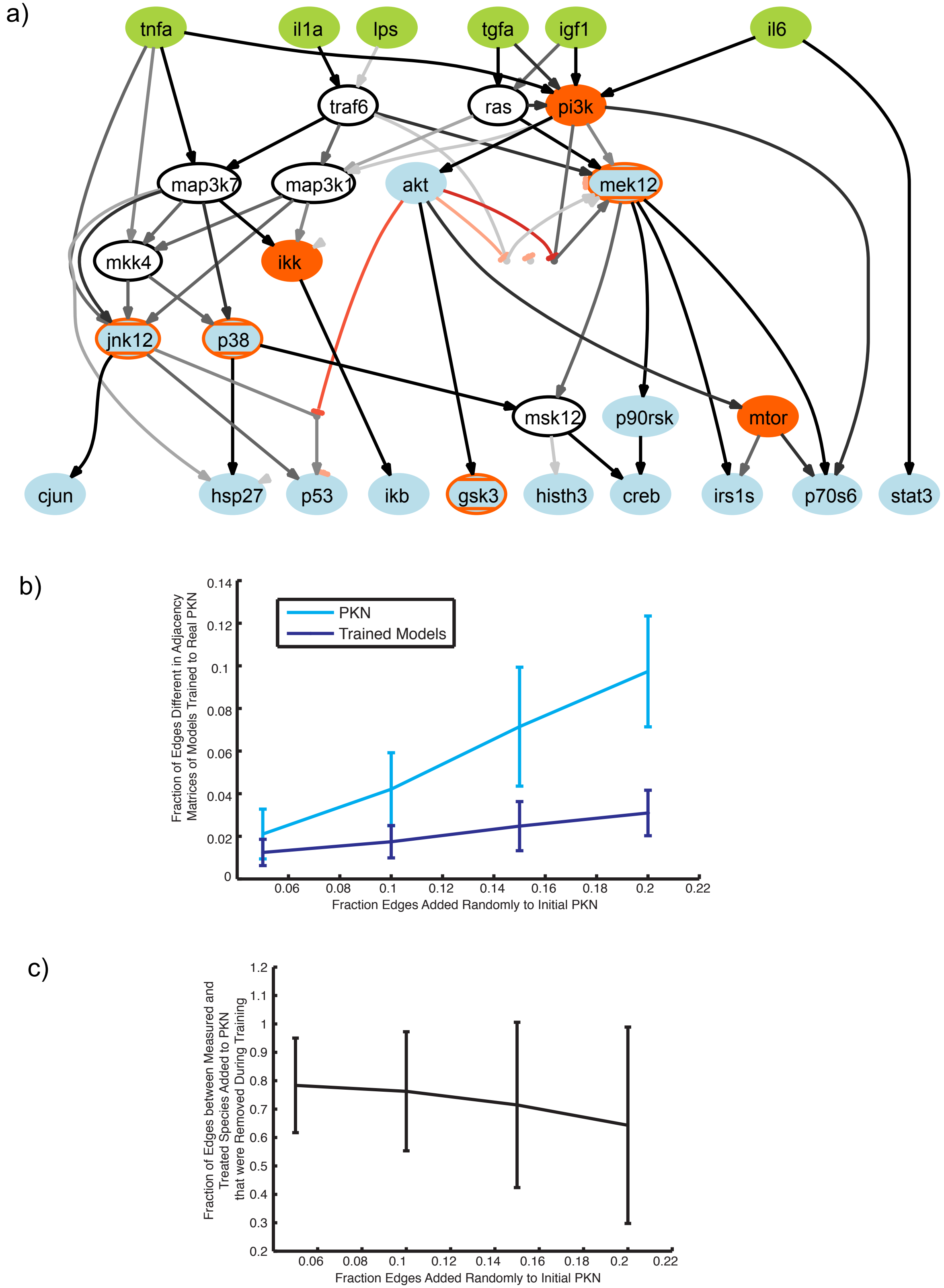
a)
tnfa
il1a
lps
tgfa
igf1
il6
traf6
ras
pi3k
map3k7
map3k1
akt
mek12
mkk4
ikk
jnk12
p38
msk12
p90rsk
mtor
cjun
hsp27
p53
ikb
gsk3
histh3
creb
irs1s
p70s6
stat3
b)
PKN
Trained Models
Fraction of Edges Different in Adjacency Matrices of Models Trained to Real PKN
Fraction Edges Added Randomly to Initial PKN
c)
Fraction of Edges between Measured and Treated Species Added to PKN that were Removed During Training
Fraction Edges Added Randomly to Initial PKN
