# Supplementary material for: Training Signaling Pathway Maps to Biochemical Data with Constrained Fuzzy Logic: Quantitative Analysis of Liver Cell Responses to Inflammatory Stimuli
Source: PLoS Comput Biol. 2011 Mar 3;7(3):e1001099. doi: 10.1371/journal.pcbi.1001099 (PMC3048376; doi:10.1371/journal.pcbi.1001099)
Supplement: Table S1 — Assessing statistical significance of cFL models derived from PKN1i. network randomization were performed. In “Swap Heads” randomization, the input of each interaction was randomly exchanged with the input of another interaction while in “Swap Tails,” this process was executed for outputs of each interaction. “Swap Inputs” randomization involved swapping the inputs of all interactions with a randomly chosen output node with the inputs of all interactions with another randomly chosen output node. Finally, completely random networks were generated with the same number of nodes and edges as the extended prior-knowledge network, at least one edge per node, and no incoming but at least one outgoing edge for each network input [29]. For the random data case, P-Values were calculated for each model trained to the real dataset using the Z-score of the model MSE compared to the distribution of randomized data models' MSEs. For the random networks case, the distribution of MSEs was not normal as assessed by the Jarque-Bera test at α≥0.001. In this case, P-value was calculated as the instance of random models with score less than that of the trained model, of which no instance was observed for any model. (0.07 MB PDF) [file pcbi.1001099.s015.pdf]

| **Randomization Method** | **Average P-Value** | **Maximum P-Value** |
|---|---|---|
| Randomize Data<br>n = 312 | $9.6 \times 10^{-68}$ | $1.8 \times 10^{-65}$ |
| Swap Heads<br>n = 1027 | $< 1.0 \times 10^{-3}$ | $< 1.0 \times 10^{-3}$ |
| Swap Tails<br>n = 1059 | $< 1.0 \times 10^{-3}$ | $< 1.0 \times 10^{-3}$ |
| Swap Inputs<br>n = 1016 | $< 1.0 \times 10^{-3}$ | $< 1.0 \times 10^{-3}$ |
| Completely Random Model<br>n = 1104 | $< 1.0 \times 10^{-3}$ | $< 1.0 \times 10^{-3}$ |
