# Supplementary material for: Training Signaling Pathway Maps to Biochemical Data with Constrained Fuzzy Logic: Quantitative Analysis of Liver Cell Responses to Inflammatory Stimuli
Source: PLoS Comput Biol. 2011 Mar 3;7(3):e1001099. doi: 10.1371/journal.pcbi.1001099 (PMC3048376; doi:10.1371/journal.pcbi.1001099)
Supplement: Table S2 — Test sets for cross validation experiment. In each test case, the measured signal under one stimulation condition with all inhibitor conditions was used as the test data. The remaining data was training data. (0.08 MB PDF) [file pcbi.1001099.s016.pdf]

	Stimulation condition	Measured signal left out
1	IGF1	GSK3
2	TGFα	GSK3
3	IGF1	Akt
4	TGFα	Akt
5	TNFα	p53
6	IL1α	p53
7	TNFα	IkB
8	IL1α	IkB
9	TGFα	CREB
10	IL1α	CREB
11	TGFα	p90RSK
12	IL1α	p90RSK
13	TGFα	Mek
14	IL1α	Mek
15	TGFα	IRS1s
16	IL1α	IRS1s
17	TGFα	p70s6
18	IL1α	p70s6
19	IGF1	p70s6
