# Supplementary material for: Training Signaling Pathway Maps to Biochemical Data with Constrained Fuzzy Logic: Quantitative Analysis of Liver Cell Responses to Inflammatory Stimuli
Source: PLoS Comput Biol. 2011 Mar 3;7(3):e1001099. doi: 10.1371/journal.pcbi.1001099 (PMC3048376; doi:10.1371/journal.pcbi.1001099)
Supplement: Table S3 — Frequency of interactions linking protein signals to phenotypic cytokine release. Frequency of links in the subset of 31 cFL models (Figure S12) with MSEs lower than one standard deviation of the family of models. (0.08 MB PDF) [file pcbi.1001099.s017.pdf]

Unprocessed Models					
**Input/Output**	**IL1B**	**IL4**	**G-CSF**	**IFNg**	**SDF1a**
**MEK1/2**	0.26	0.29	0.29	0.32	0.39
**IkB**	0.84	0.90	0.61	0.77	0.10
**STAT3**	1.00	1.00	0.94	1.00	0
**GSK3**	0.29	0.29	0.16	0.39	0.52
**CREB**	0.26	0.19	0.23	0.16	0.29
**c-Jun**	0.48	0.55	0.58	0.58	0.64
**Hsp27**	0.58	0.65	0.71	0.45	0.74
**Filtered Models**					
**Input/Output**	**IL1B**	**IL4**	**G-CSF**	**IFNg**	**SDF1a**
**MEK1/2**	0.19	0.19	0.19	0.26	0.32
**IkB**	0.84	0.87	0.61	0.74	0.10
**STAT3**	1.00	1.00	0.94	1.00	0
**GSK3**	0.26	0.13	0.13	0.29	0.39
**CREB**	0.19	0.13	0.19	0.06	0.26
**c-Jun**	0.42	0.45	0.52	0.48	0.52
**Hsp27**	0.55	0.52	0.61	0.39	0.71
