# Supplementary material for: Training Signaling Pathway Maps to Biochemical Data with Constrained Fuzzy Logic: Quantitative Analysis of Liver Cell Responses to Inflammatory Stimuli
Source: PLoS Comput Biol. 2011 Mar 3;7(3):e1001099. doi: 10.1371/journal.pcbi.1001099 (PMC3048376; doi:10.1371/journal.pcbi.1001099)
Supplement: Figure S5 — Filtered cFL network models derived from training PKN1 processed to include two-input AND gates (PKN1a) to HepG2 dataset. The PKN1 (Figure S2a) was processed to include all two-input AND gates (Figure S2c) and CellNOpt-cFL used to train 191 network models to the HepG2 dataset. Reduction of family of cFL models indicates that cFL AND gates can be removed without greatly affecting the resulting refined model score (a and b; b is a portion of the graph shown in a). The structures of the family of cFL network models trained to the HepG2 dataset are shown (c). Links colored black were present in all models whereas links colored grey were present in a fraction of the models (a darker grey indicates that the cFL gate was present in more models). Filtered cFL network models are shown. Fit to experimental data (d) is displayed as described in Figures S1 and S3. Plots were generated by CellNOpt. Note that, when this PKN1a is used to train the networks, most trained models include the Ras → Map3k1 cFL gate. The inclusion of this link is in contrast to the models obtained when two-input AND gates are only included for inhibitory interactions (Figure 5, Figure S6), where only a few models include this link. This difference is also reflected in the fact that cFL network models processed to include all two-input AND gates are better able to fit data describing c-Jun activation under TGFα stimulation (d compared to Figures S7 and S8). Graphs of cFL network models were generated a CellNOpt routine using the graphviz visualization engine (www.graphviz.org) followed by manual annotation in Adobe Illustrator. (0.27 MB PDF) [file pcbi.1001099.s005.pdf]

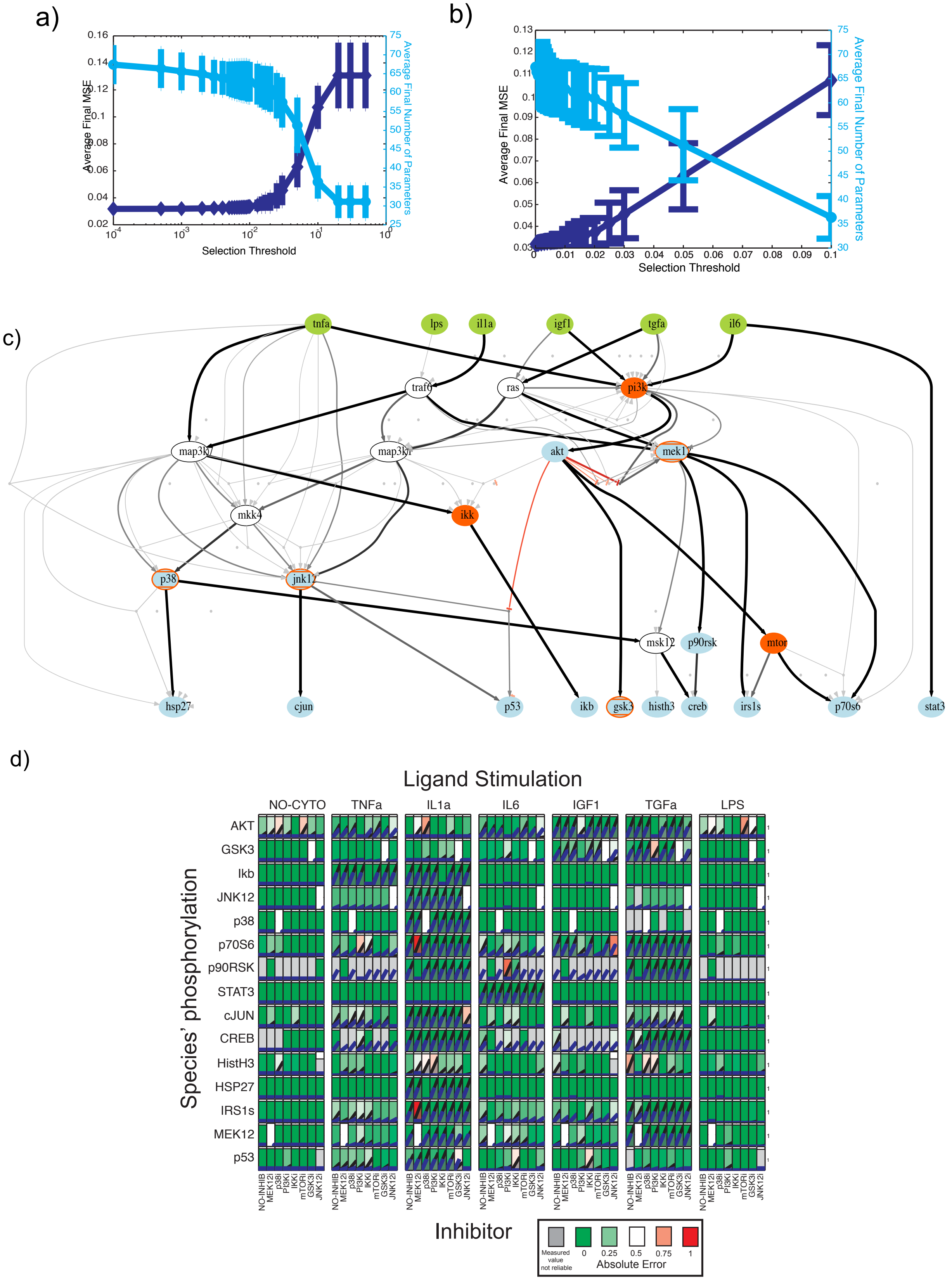
a)
Average Final MSE
Average Final Number of Parameters
Selection Threshold
b)
Average Final MSE
Average Final Number of Parameters
Selection Threshold
c)
tnfa
lps
il1a
igf1
tgfa
il6
traf6
ras
pi3k
map3k7
map3k1
akt
mek12
mkk4
ikk
p38
jnk12
msk12
p90rsk
mtor
hsp27
cjun
p53
ikb
gsk3
histh3
creb
irs1s
p70s6
stat3
d)
Ligand Stimulation
NO-CYTO
TNFa
IL1a
IL6
IGF1
TGFa
LPS
Species' phosphorylation
AKT
GSK3
Ikb
JNK12
p38
p70S6
p90RSK
STAT3
cJUN
CREB
HistH3
HSP27
IRS1s
MEK12
p53
NO-INHIB
MEK12i
p38i
PI3Ki
IKKi
mTORi
GSK3i
JNK12i
Inhibitor
Measured value not reliable
0
0.25
0.5
0.75
1
Absolute Error
