# Supplementary material for: Training Signaling Pathway Maps to Biochemical Data with Constrained Fuzzy Logic: Quantitative Analysis of Liver Cell Responses to Inflammatory Stimuli
Source: PLoS Comput Biol. 2011 Mar 3;7(3):e1001099. doi: 10.1371/journal.pcbi.1001099 (PMC3048376; doi:10.1371/journal.pcbi.1001099)
Supplement: Figure S10 — Fit of PLSR model of phenotypic cytokine release data. A three-component PLSR model fit normalized cytokine release data well in most cases except the condition of TNFα stimulation and Iκb inhibition. The data is displayed as described in and S3. Plots were generated by CellNOpt. (0.13 MB PDF) [file pcbi.1001099.s010.pdf]

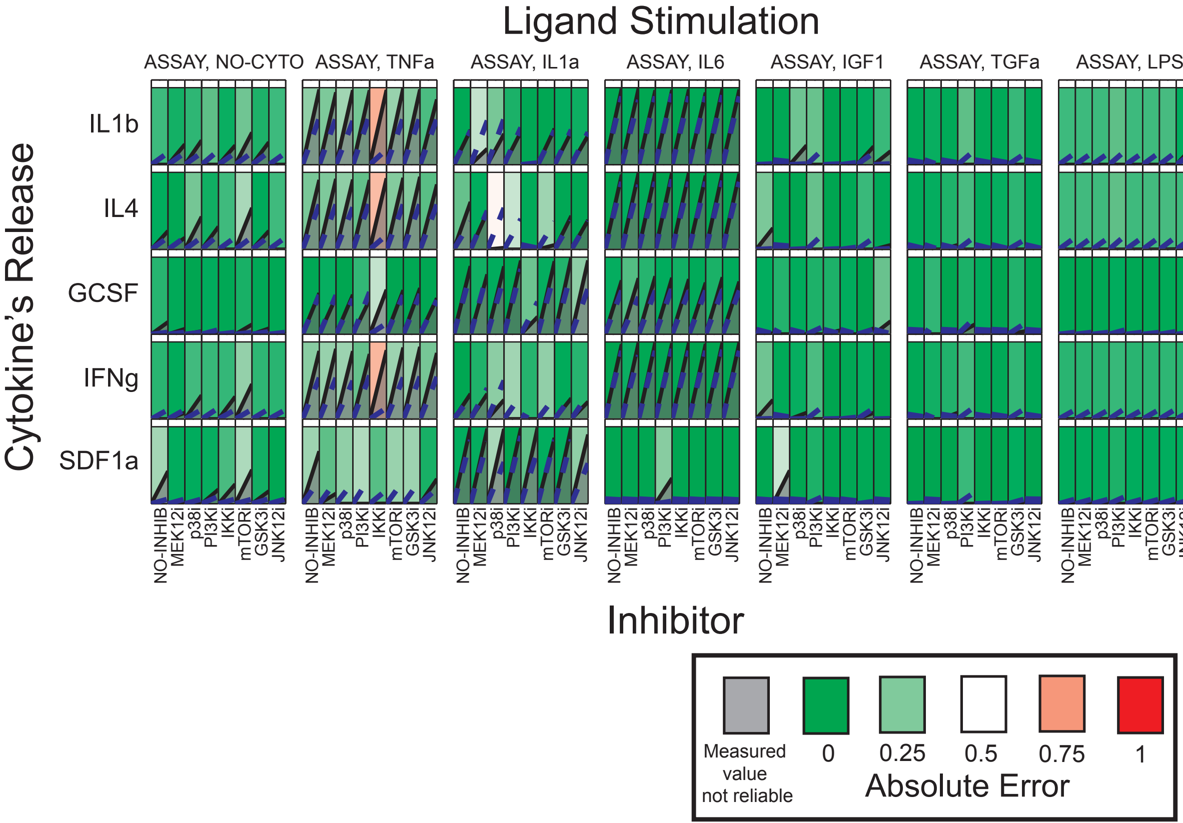

Ligand Stimulation
ASSAY, NO-CYTO
ASSAY, TNFa
ASSAY, IL1a
ASSAY, IL6
ASSAY, IGF1
ASSAY, TGFa
ASSAY, LPS
Cytokine's Release
IL1b
IL4
GCSF
IFNg
SDF1a
1
1
1
1
1
NO-INHIB
MEK12i
p38i
PI3Ki
IKKi
mTORi
GSK3i
JNK12i
Inhibitor
Measured value not reliable
0
0.25
0.5
0.75
1
Absolute Error
