# Supplementary material for: Training Signaling Pathway Maps to Biochemical Data with Constrained Fuzzy Logic: Quantitative Analysis of Liver Cell Responses to Inflammatory Stimuli
Source: PLoS Comput Biol. 2011 Mar 3;7(3):e1001099. doi: 10.1371/journal.pcbi.1001099 (PMC3048376; doi:10.1371/journal.pcbi.1001099)
Supplement: Table S4 — Experimentally verified and computationally predicted transcription factor binding sites in relevant genes. Genes were queried in BioBase TRANSFAC [62], [63] for experimentally verified or computationally predicted transcription factor binding sites and the March 2006 (NCBI36/hg18) assembly of UCSC Genome Bioinformatics (http://genome.ucsc.edu/) for computationally predicted transcription factor binding sites. Those binding sites listed below were included either because they were binding sites of phosphorylated proteins measured or transcription factors modulated by phosphorylated proteins measured. (0.09 MB PDF) [file pcbi.1001099.s018.pdf]

Gene	Transcription Factor Binding Site
IL4	IRF1[*+]<br>IRF2[*]
IL1B	NFκB[+]<br>CREB[*]<br>C/EBPB[*]<br>STAT1[*¥]
G-CSF	KFκB[*+]<br>C/EBPα[*‖]<br>C/EBPB[*]<br>TAL1α/E47[+§]
SDF1α	STAT[+]<br>TAL1α/E47[+§]<br>c-Jun[#]<br>STAT1[#¥]
IFNγ	IRF1[+]<br>IRF2[+]<br>STAT3[*]<br>NFκB[*]<br>CREB[*]<br>c-Jun[*]
IL6	NFκB[*]<br>AP-1[*]<br>c-Jun[*]<br>C/EBPB[*]<br>CREB[+]<br>IRF1[+]<br>IRF2[+]
IRF1	STAT[+*]<br>AP-1[+]<br>NFκB[*]
IRF2	CREB[+]<br>IRF1[*]

[*] denotes transcription factor binding site (BioBase TRANSFAC)
[#] denotes transcription factor binding site in the vicinity of the gene (BioBase TRANSFAC)
[+] denotes site computationally predicted (UCSC Genome Bioinformatics)
[¥] STAT1 and STAT3 have similar binding motifs (BioBase TRANSFAC)
[‖] C/EBPα is phosphorylated by GSK3 [1]
[§] TAL1α is phosphorylated by Erk1 [2] and Akt [3] (REF). Phosphorylation by Akt inhibits TAL1α repressor activity [3].

References

1. Ross S, Erickson R, Hemati N, MacDougald O (1999) Glycogen synthase kinase 3 is an insulin-regulated C/EBPalpha kinase. Mol Cell Biol 19: 8433-8441.
2. Cheng J, Cobb M, Baer R (1993) Phosphorylation of hte TAL1 oncoprotein by the extracellular-signal-regulated protein kinase ERK1. Mol Cell Biol 13: 801-808.
3. Palamarchuk A, Efanov A, Maximov V, Aquilan R, Croce C, et al. (2005) Akt phosphorylates Tal1 oncoprotein and inhibits its repressor activity. Cancer Res 65: 4515-4519.
