# Supplementary material for: Training Signaling Pathway Maps to Biochemical Data with Constrained Fuzzy Logic: Quantitative Analysis of Liver Cell Responses to Inflammatory Stimuli
Source: PLoS Comput Biol. 2011 Mar 3;7(3):e1001099. doi: 10.1371/journal.pcbi.1001099 (PMC3048376; doi:10.1371/journal.pcbi.1001099)
Supplement: Text S1 — Supplementary materials and methods. (0.09 MB PDF) [file pcbi.1001099.s019.pdf]

Supplementary Methods

HepG2 training and follow-up data phospho-ERK measurement inconsistencies

In the training dataset, ERK was phosphorylated solely under one stimulatory condition (TGFα) and did not increase with increasing MEK phosphorylation. When compared the raw data between this and the follow-up dataset discussed below, ERK did indeed respond in many other conditions in the initial training dataset, but signal intensity was much lower in this experiment, causing many ERK phosphorylation measurements to be below the noise threshold and recorded as zero during the normalization process. Because of these discrepancies with the phospho-ERK measurements, we did not include it in our training dataset.

Constrained fuzzy logic

Prior to implementing constrained fuzzy logic for network training, we investigated the use of Mamdani [1] and Sugeno [2] fuzzy logic gates with varying number and functional forms of membership functions. The cFL framework we use in this work represents each biological interaction with Sugeno gates with normalized Hill input membership functions and constant output membership functions of zero and one. Each AND gate is a fuzzy logic rule with an AND operator of "min." In this formalism, OR gates are evaluated by the "max" defuzzification method that operates on the outputs of fuzzy logic rules.

The use of normalized Hill functions assumes that species reach the same level of saturation under activation by any of its possible inputs. Biologically, this assumption does not always hold. However, during our initial methods development, we deemed this assumption acceptable as the use of the normalized Hill function did not cause any noticeable issues during the model training and allowed each parameter to have a distinct meaning with the sensitivity parameter, k, specifying EC_{50} and the Hill coefficient, n, specifying sharpness of transition.

Simulation

A set of functions was implemented in MATLAB (Mathworks, Inc.) and integrated into CellNetOptimizer to convert BL models to cFL models and determine the logic steady state of node states of a given cFL network under given experimental conditions. To calculate the logic steady state, nodes of the network are updated until they reach a stable state. If the network contains negative feedback, a logic steady state cannot be computed, similar to the Boolean case [3]. Penalization of not-computed stated states leads then to the absence of negative feedback in resulting models [4]. To increase the efficiency of the training process, in the HepG2 prior knowledge network we determined the negative interactions that would result in negative feedback in CellNOpt using the MATLAB (Mathworks, Inc.) software CellNetAnalyzer [5], and we removed them prior to optimization.

Model Refinement

Model parameters were refined using the MATLAB active-set algorithm, a Sequential Quadratic Programming method for nonlinear constrained optimization (http://www.mathworks.com/access/helpdesk/help/toolbox/optim/ug/brnoxzl.html).

PLSR model of cytokine release data

Luminex data describing release of 50 cytokines at time zero and three hours after stimulation was examined. Twenty cytokines were chosen to model based on the consistency and reliability of the data (e.g. if the data was grossly inconsistent under similar experimental treatment conditions, it was not considered). Data for these cytokines were normalized similarly to the phospho-signal dataset except no data was considered below the lower level of detection because it had already been filtered for consistency [4].

A preliminary three-component PLSR model was constructed using DataRail [6] by regressing the normalized cytokine release data against the signaling data. Five cytokines (IL1B, IL4, G-CSF, IFNγ, and SDF1α) were chosen for further study based on the criteria that the R^2 values of the PLSR model for those cytokines be greater than 0.70.

Further analysis suggested that cytokine measurements with lower R^2 values were not robust (i.e. varied in measured value even under similar stimulation and inhibition conditions).

A new PLSR model was then generated with DataRail by regressing the normalized cytokine release data against the signaling data. Three components were chosen to be optimal by seven-fold cross-validation.

Linking prior knowledge network to cytokine release nodes

The clustering of the protein signals in principle components space of both a principle component model of the signals as well as the principle components of the PLSR model was considered when choosing signaling nodes to link to the cytokine release nodes. If protein signals clustered together consistently, the signal most downstream in the prior knowledge networks was chosen. Based on this analysis, the following protein signaling nodes were linked to each cytokine release node: MEK1/2, CREB, GSK3, c-Jun, Hsp27, IκB, and STAT3.

References

1. Mamdani E (1976) Application of fuzzy logic to approximate reasoning using linguistic synthesis. Proc 6th Internat Symp Mult-Val Logic, IEEE 76CH1111-4C: 196-202.
2. Sugeno M, Nishida M (1985) Fuzzy control of a model car. Fuzzy Sets and Systems 16: 103-113.
3. Klamt S, Saez-Rodriguez J, Lindquist J, Simeoni L, Gilles ED (2006) A Methodology for the Structural and Functional Analysis of Signaling and Regulatory Networks. BMC Bioinformatics 7: 56.
4. Saez-Rodriguez J, Alexopoulos L, Epperlein J, Samaga R, Lauffenburger D, et al. (2009) Discrete logic modelling was a means to link protein signalling networks with functional analysis of mammalian signal transduction. Molecular Systems Biology 5.
5. Klamt S, Saez-Rodriguez J, Gilles ED (2007) Structural and functional analysis of cellular networks with CellNetAnalyzer. BMC Systems Biology, 1:2.
6. Saez-Rodriguez J, Goldsipe A, Muhlich J, Alexopoulos L, Millard B, et al. (2008) Flexible Informatics for Linking Experimental Data to Mathematical Models via DataRail. Bioinformatics 24: 840-847.
